# Foliar application of magnesium and the use of plant growth-promoting rhizobacteria improve photosynthetic physiology and the yield components of soybean

**DOI:** 10.3389/fpls.2025.1694929

**Published:** 2026-01-20

**Authors:** Nelson Câmara de Souza Júnior, Vitória Almeida Moreira Girardi, Maiara Luzia Grigoli Olivio, Thalita Fischer Santini Mendes, Aline Marchetti Silva Matos, Naiane Antunes Alves Ribeiro, Bárbara Pereira Christofaro Silva, Fernando Shintate Galindo, Liliane Santos de Camargos, Marcelo Andreotti

**Affiliations:** 1 Department of Plant Health, Rural Engineering and Soils, São Paulo State University (UNESP), School of Engineering, Ilha Solteira, São Paulo, Brazil; 2 Department of Biology and Animal Science, São Paulo State University (UNESP), School of Engineering, Ilha Solteira, São Paulo, Brazil; 3 Departament of Plant Production, College of Agricultural and Technological Sciences, São Paulo State University - UNESP - FCA, Dracena, São Paulo, Brazil

**Keywords:** *Bacillus subtilis*, glycine max, magnesium supply, metabolic improvement, photosynthesis, plant physiology, *Priestia megaterium*

## Abstract

**Introduction:**

The study evaluated the effects of foliar magnesium (Mg) application and inoculation/co-inoculation with plant growth-promoting rhizobacteria (PGPRs) on soybean physiological metabolism and yield in a long-term no-tillage system under Brazilian Cerrado conditions. Despite the relevance of PGPRs for crop resilience, there remains a research gap regarding the use of *Priestia megaterium* and *Bacillus subtilis* as stimulators of plant physiological metabolism under field conditions, particularly their potential to mitigate adverse climate-related stresses. The novelty of this study lies in the unprecedented assessment of the interaction between foliar Mg application and the inoculation of these bacteria in soil under a long-term no-tillage system. We investigate how this combination can enhance the crop’s physiological metabolism, contributing to refined agricultural management techniques that are essential for addressing the challenges posed by climate change.

**Methods:**

The field experiment was carried out in an Oxisol over two growing seasons, using a randomized complete block design in a factorial scheme (3 x 2), combining three inoculation treatments (*Bradyrhizobium*, *Bacillus subtilis*, and *Priestia megaterium*) with or without foliar Mg application at the V_6_ soybean growth stage.

**Results:**

The control treatment (*Bradyrhizobium japonicum*) showed higher ureide content (approximately 25% greater than the seed co-inoculation treatment) and greater plant height in both growing seasons. Principal Component Analysis (PCA) indicated that the control was more susceptible to heat stress, as evidenced by higher MDA and peroxide levels. Furrow co-inoculation exhibited intermediate performance, with greater sensitivity to high temperatures in the 2023/24 season and lower water-use efficiency in 2024/25. In contrast, seed co-inoculation increased the number of pods (40%) and grains per plant (45%), enhanced gross photosynthesis (40%), instantaneous water-use efficiency (25%), and internal carbon concentration (10%), compared with the control in both evaluated seasons.

**Conclusion:**

The combination of seed co-inoculation with foliar Mg application reduced hydrogen peroxide content, suggesting mitigation of reactive compounds and greater physiological stability. In summary, seed co-inoculation associated with foliar Mg application improved physiological attributes and yield components of soybean under field conditions in the Cerrado.

## 1 Introduction

Magnesium (Mg) plays an important role in plant physiological processes, as it is the central ion of the chlorophyll molecule. Furthermore, it is crucial for the synthesis of enzymes such as Ribulose-1,5-Bisphosphate Carboxylase/Oxygenase (Rubisco) and Phosphoenolpyruvate Carboxylase (PEPcase), which are vital for the photosynthetic process, in addition to being an essential element for the production of adenosine triphosphate (ATP) (Taiz and Zeiger, 2016). All these factors make magnesium fertilization essential to guarantee productivity in C_3_ and C_4_ metabolism plants. Studies with foliar applications of Mg in stages where root absorption efficiency begins to decline have shown that the application of the nutrient can provide productivity gains due to the improvement of the plants photosynthetic apparatus (Wang et al., 2020). Since Mg helps in vital photosynthetic processes by enabling Rubisco to act as a carboxylase, and considering that about 85% of plant species on the planet are C_3_ plants, which are highly prone to stress losses (Taiz and Zeiger, 2016), research on this nutrient is indispensable for scientific innovation projects aimed at refining agricultural production.

Even in systems with consolidated no-tillage for years, results show that crops such as soybean and maize, when receiving foliar applications of Mg, exhibited a sort of “stimulating effect,” where photosynthetic parameters were positively affected, leading to considerable productivity gains (Altarugio et al., 2017; Branquinho, 2020; Rodrigues et al., 2021). With the growing global demand for food, coupled with climate change, techniques that provide production gains are becoming more and more important and deserve attention in global scientific research.

Heat waves and severe droughts have affected the productive potential of crops, which can only be achieved if all necessary conditions (water, light, temperature, and nutrition) for development are met. Research aimed at mitigating abiotic stresses in crops is crucial, as such stresses can negatively affect food security. Water deficits and extreme temperatures are uncontrollable climatic factors present in the lives of farmers. This becomes essential since, in the field, it is impossible to predict the climatic conditions to which the plant will be subjected in the field, due to the climatic variations observed in each cultivation year. With imminent climate change, research data indicate that these uncontrollable factors could lead to a reduction in Brazilian production by around 10% to 40% by 2050 (Ministério da Ciência, Tecnologia e Inovação Brasil, 2020). This data is extremely important because Brazil is one of the key countries for global food production. High temperatures cause direct damage to plants, leading to tissue overheating, which can result in indirect effects such as water deficits, potentially reducing cell turgor (Pretty and Bharucha, 2014; Chatterjee et al., 2019).

The plant’s response to any type of stress will vary from species to species. However, as soon as the plant detects any stressor, it produces a metabolic response known as reactive oxygen species. Thus, concerning abiotic stresses, the main reflection in the plant is a decrease in its metabolism, leaf curling, lower photosynthetic rates, the development of cuticles and trichomes on the leaf surface to minimize water loss, with the primary goal of completing its life cycle (Taiz and Zeiger, 2016; Broetto et al., 2017). Therefore, strategies aimed at improving the photosynthetic metabolism of these plants are indispensable due to the increasing number of stress factors resulting from climate change. Extreme heat waves, for example, caused the planet’s average temperature to rise by about 1.45°C in 2023 (United Nations (ONU), 2024). These data highlight the challenges faced by global food production, as each agricultural year brings different climatic factors that affect crops in the field (Chatterjee et al., 2019). Stimulating the physiological metabolism of plants in the field appears to be a viable alternative to help them cope with the adversities imposed by uncontrollable climatic factors, such as water deficits and temperature fluctuations. Field research on plant metabolism stimulants is important because it can contribute to ensuring global food security. Even though it is impossible to predict or fully understand the climatic dynamics of each agricultural year, it is believed that physiological stimulants may be beneficial and could become a viable option for agricultural production. Among these, the use of plant growth-promoting bacteria and nutrients such as nitrogen (N), Mg, among others, stand out (Broetto et al., 2017; Poudel et al., 2021; Romero-Munar et al., 2023).

To assist in the improvement of photosynthetic physiology, another common ally used in agriculture is plant growth-promoting rhizobacteria (PGPR), which assist in root development and growth, improving soil exploration and benefiting the absorption of water and nutrients (Poudel et al., 2021; Armanhi et al., 2021). In soybean, the genus *Bradyrhizobium* is already commonly used due to its crucial role in biological nitrogen fixation (BNF), which significantly increases crop production parameters, making Brazilian soybeans highly productive without the need for nitrogen fertilizer (Hungria et al., 2001; Hungria and Nogueira, 2020). Brazil has taken on an important role in the global use of *Bradyrhizobium*, making Brazilian soybean highly competitive, with a reduction of U$ 13 billion in nitrogen fertilizer use (Hungria and Nogueira, 2020; Hungria et al., 2020). This technique is highly effective, sustainable, and low-cost, making the use of the bacterium a common practice among soybean producers in the country.

Bacteria from the genera *Bacillus* and *Priestia* also stand out as PGPRs and are commonly used in co-inoculation practices (mixtures of different bacterial species and genera). They help plants defend against biotic stresses, such as pathogen and pest attacks, as well as abiotic stresses, including tolerance to water deficits. These bacteria also promote plant growth and nutrient absorption, phytohormone synthesis, siderophore production, and improvements in soil microbiological conditions and fertility, aiding in nutrient solubilization (Kumar et al., 2009; Duca, 2014; Taha, 2016; Ribeiro et al., 2018). Studies combining species of *Priestia megaterium* and *Bacillus subtilis* have observed productivity gains in crops, explained by greater solubilization of non-labile phosphorus by the bacteria and a well-developed root system capable of increasing root hairs, which improves nutrient absorption (Furmam, 2019; Franchi, 2022). Co-inoculation can broaden the metabolic efficiency spectrum in the crop, and the use of *Bacillus* spp. in combination with *Bradyrhizobium* in soybean has shown significant results in terms of yield increase, demonstrating that this technique is both effective and viable. Studies involving the co-inoculation of *Bacillus/Priestia* and *Bradyrhizobium* have shown yield increases of up to 840 kg ha^-1^ of soybean grains (Paiva et al., 2020; Sousa et al., 2021; Oliveira-Paiva et al., 2024).

Soybean is one of the most prominent agronomic crops in Brazil and worldwide, and according to data from the Conab – Companhia Nacional de Abastecimento (2024), the estimated production of the 2023/24 Brazilian soybean crop reached 147.35 million tons, a decrease of approximately 7 million tons, reflecting the effects of abiotic stresses, mainly water deficits due to a lack of rainfall, and heat waves from November to February during the production cycle. Brazil is currently the world’s largest soybean producer, and according to CONAB’s report, the data also states that soybean is one of the crops most prone to stress issues, precisely because it is a C_3_ plant, highly influenced by photoperiod (Taiz and Zeiger, 2016). Therefore, due to its importance in global agriculture, strategies aimed at increasing production sustainably are indispensable to ensure global food security, in addition to being well received by consumers and producers.

The hypothesis of this research is that Mg, when combined with the co-inoculation of PGPRs, can contribute to improving the photosynthetic metabolism of soybean, resulting in visible benefits even in soils with high magnesium content and adequate base saturation. This optimization of photosynthesis benefits the plant by enabling it to better withstand adverse field conditions, such as the occurrence of high temperatures during crop development, thereby conferring tolerance to such conditions and allowing productivity gains. The use of Mg and PGPRs may represent an alternative strategy to minimize losses caused by climate change, which threaten food security and can negatively impact agricultural production. Considering these points, the objective of this study was to evaluate the effect of foliar Mg fertilization and the use of PGPRs, both individually and in combination, in a soil under a well-established NTS with no nutritional limitations, as well as their effects on stimulating photosynthetic physiology and soybean grain yield under the conditions of the Brazilian Cerrado.

## 2 Methods

### 2.1 Characterization of the experimental area and climate

**Figure 1:**
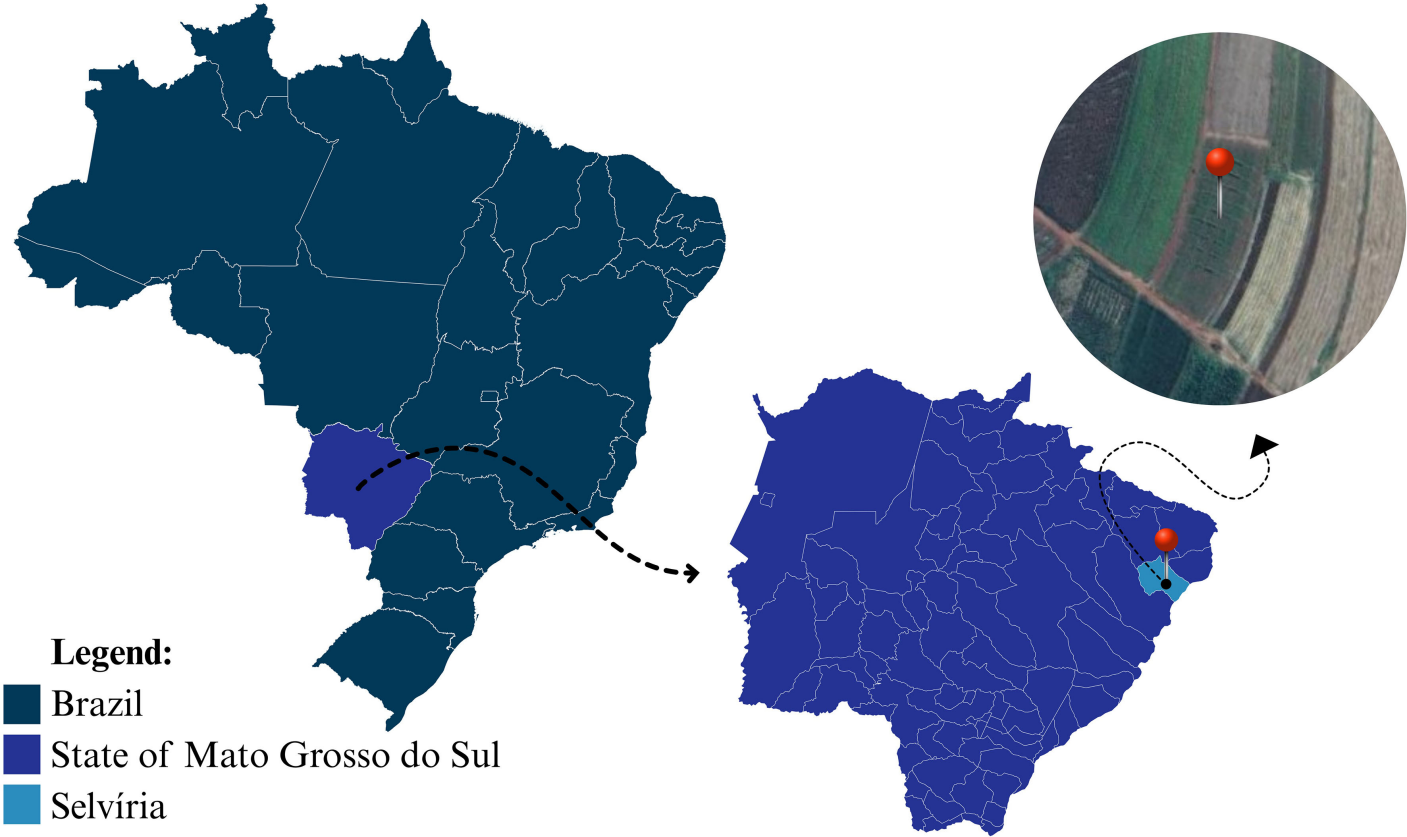
Location of the experimental area in the municipality of Selvíria, State of Mato Grosso do Sul, Brazil.

The research project was conducted in the field at the Teaching, Research, and Extension Farm (FEPE) of the São Paulo State University “Júlio de Mesquita Filho” - Ilha Solteira Engineering Faculty (UNESP-FEIS), located in the city of Selvíria, MS, at geographical coordinates 20°18’S and 51°22’W, with an altitude of 370 m above sea level (**Figure 1**).

**Figure 2:**
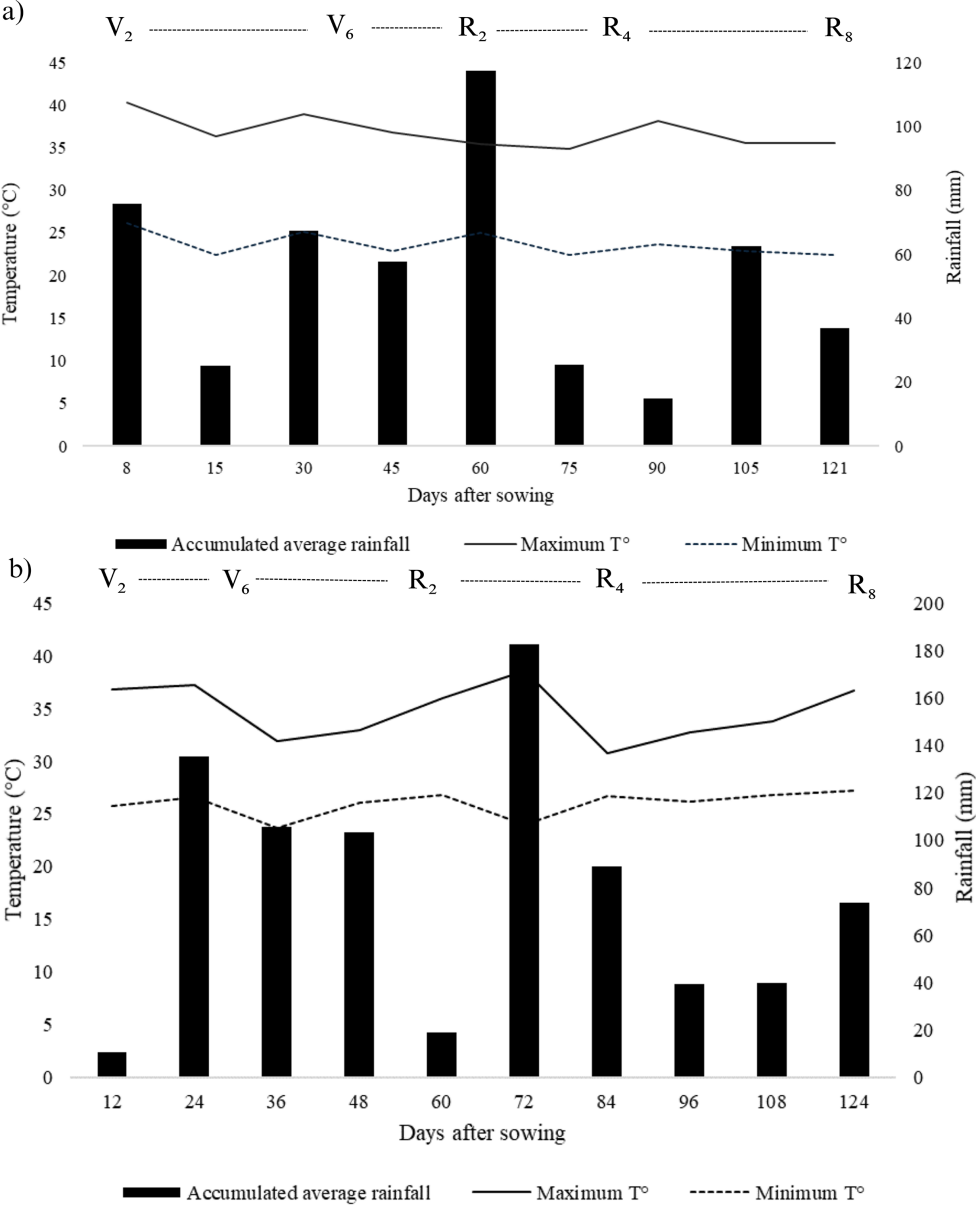
Climatic data for maximum and minimum temperature and rainfall in the municipality of Selvíria, State of Mato Grosso do Sul, Brazil, for the 2023/24 **(a)** and 2024/25 **(b)** crop seasons, during the experiment period. Temperature and rainfall data, Clima Unesp (2023/2024 and 2024/25). V_2_, a fully developed trifoliate leaf, characterized by the development of the second trifoliate; V_6_, development of the sixth trifoliate leaf on the plant’s main stem; R_2_, full flowering; R_4_, full pod formation stage; R_8_, physiological maturity (harvest maturity stage).

According to the Köppen and Geiger (1928) international classification, it can be stated that the climate of the municipality of Selvíria presents the subtypes Aw – humid tropical, mesothermal, with a dry winter and hot summer, and irregular annual rainfall distribution. The experimental area can be considered flat, and the rainfall indices in the region range from 1200 to 1500 mm per year. The climatological graph for the period of the experiment during the 2023/24 and 2024/25 crop seasons is presented in **Figure 2**.

The soil of the experimental area was characterized as dystrophic Red Latosol (Oxisol) (52% clay) (Santos et al., 2018). In the area where the project was carried out, soil samples were collected before soybean planting to evaluate its fertility (**Table 1**).

**Table 1:** Chemical characterization of the soil in the 0.00-0.20 layer in Selvíria, State of Mato Grosso do Sul, Brazil, for the 2023/24 and 2024/25 crop seasons.

Crop	Layer m	P_resin_	OM	pH	K	Ca	Mg	H+Al	Al	SB	CEC	V%
		mg dm^-3^	g dm^-3^	CaCl_2_	mmol_c_ dm^-3^							
2023/24	0-0.20	50	24	5.3	2.5	32	23	34	0	57.5	91.5	63
2024/25	0-0.20	63	24	5.4	3.9	37	24	28	0	64.9	92.9	70

P, Phosphorus; MO, soil organic matter; pH, acidity; K, potassium; Ca, calcium; Mg, magnesium; H+Al, potential acidity; V%, base saturation; Al, aluminium.

The experimental area in question has a history of 13 years of using the no-tillage system, with experiments installed annually using conservationist methods (maintenance of straw, crop rotation), where the preceding crop was grain sorghum (*Sorghum bicolor*) intercropped with Piatã grass (*Urochloa brizantha*).

### 2.2 Installation and conduct of the experiment

In the last week of October 2023 and 2024, the weeds were desiccated with the herbicide glyphosate (1.56 kg ha^-1^ of active ingredient), and soybean planting was carried out on November 16, 2023, and November 14, 2024. The cultivar used in both growing seasons was 80HO190 IPRO, which has an indeterminate growth habit, high yield potential, and lodging resistance. Row spacing was set at 0.50 m, with a seeding rate of 14.5 seeds per meter, targeting a plant population of 220,000 plants ha^−1^ (assuming 80% germination). The seeding depth was approximately 0.05 m.

The application of nitrogen-fixing and growth-promoting bacteria followed the recommendations for each product (*Bradyrhizobium* and *Bacillus*/*Priestia*). For inoculation with *Bradyrhizobium japonicum* (strains Semia 5079 and 5080) via seeds, a liquid inoculant was used at the dose recommended by the supplier (Soybean – 2.0 x 10^9^ CFU mL^-1^). For co-inoculation with *Bacillus* and *Priestia* (the combined application of BRM 119 (*P. megaterium*) and BRM 2084 (*B. subtilis*)), a commercial product was used (composed of a mixture of the two PGPB, *B. subtilis* and *P. megaterium*, at a concentration of 4.0 × 10^9^ CFU mL^−1^), applied at the manufacturer’s recommended dose for seed treatment and at twice the commercial dose for furrow application (100 mL via seed and 200 mL via furrow). The commercial products were applied to the seeds in the shade immediately before sowing; for furrow spraying, the application was carried out simultaneously with the sowing operation conducted in the morning.

The experiment was conducted in a randomized block design, in a 3x2 factorial scheme with 4 repetitions, totaling 24 experimental plots. Three treatments with bacteria were evaluated (combination of *B. subtilis* and *P. megaterium* on seeds + *Bradyrhizobium* on seeds; *Bradyrhizobium* on seeds + combination with *B. subtilis* and *P. megaterium* in the sowing furrow, and only *Bradyrhizobium* on seeds), with or without foliar Mg spraying. The seeds were pre-treated with a commercial product containing pyrazole, strobilurin, and benzimidazole, using 100 mL of the commercial product for every 50 kg of seeds. The product chosen for Mg supply was magnesium sulfate (MgSO_4_), at a standard dose of 8 kg ha^-1^, adjusted from different studies in the literature (Deliboran et al., 2011; Branquinho, 2020; Rodrigues et al., 2021). For sowing fertilization, 300 kg ha^-1^ of the 08-28-16 (N-P_2_O_5_-K_2_O) formulation was used.

**Figure 3:**
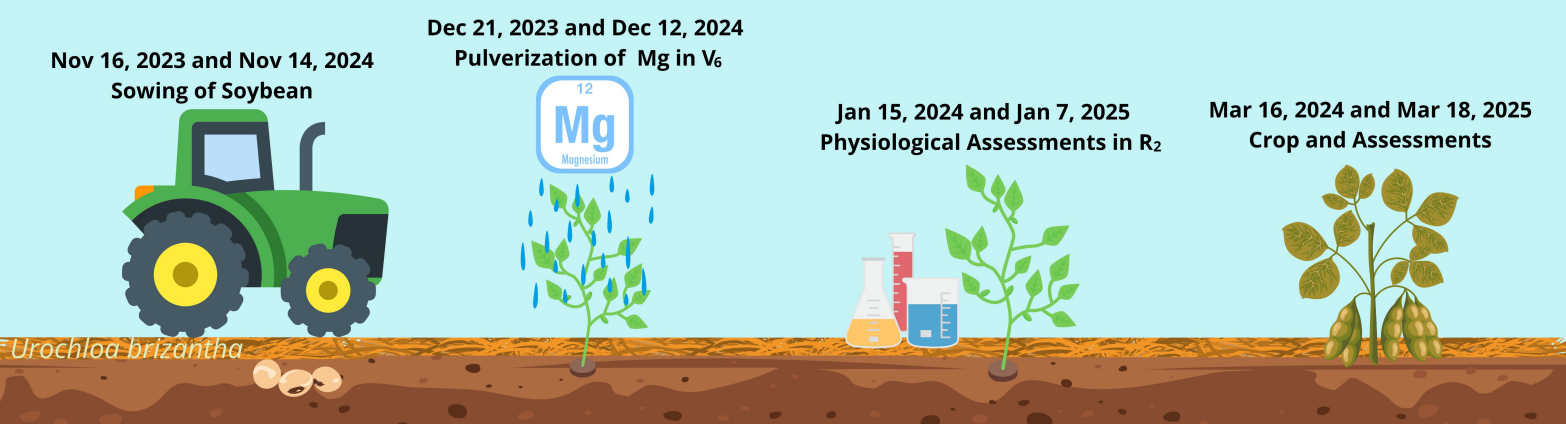
Management, application, and evaluations in the soybean crop in the municipality of Selvíria, State of Mato Grosso do Sul, Brazil, during the 2023/24 and 2024/25 crop seasons. V_6_, when the sixth node of the plant is fully expanded with trifoliate leaves; R_2_, full flowering.

The Mg application was carried out at the V_6_ stage (six fully developed nodes) (**Figure 3**), as from the V_5_ stage it is already possible to determine the potential number of nodes the plant may present, along with a considerable increase in metabolic rates. The physiological evaluations of the crop were performed at the R_2_ stage (full flowering), considering that this stage represents the peak of flowering, when the need for chlorophyll for photosynthesis is greatest. For soybean phenology, V_6_ is characterized by the presence of six fully developed nodes bearing trifoliate leaves. This stage is considered ideal for foliar nutritional management in soybean crops, as the plant exhibits active metabolic processes and reaches an approximate height of 30 to 35 cm. The physiological evaluations were conducted at the R_2_ stage, corresponding to full flowering. At this stage, the plant is metabolically active, with intensified photosynthetic activity directed toward the initial development of pods. To avoid masking the real effect of Mg, elemental sulfur (S) was applied by foliar spraying on the same day in the plots without MgSO_4_ application, using the same dose as the MgSO_4_.

Each experimental plot consisted of 16 rows, each 10 meters long, totaling 80 m² per plot. For weed and pest control, herbicides and insecticides were applied according to field observations. The harvest of the 24 experimental plots for evaluation was carried out on 03/16/24 and 03/18/25. On the same day of harvest in both crop seasons, evaluations of production components and grain yield were conducted.

### 2.3 Crop assessments

#### 2.3.1 Assessments physiological

When the soybean plants were at the R_2_ phenological stage, with the third newly expanded leaf of the main stem, gas exchanges were evaluated in each plot in the morning (from 7 to 11 am) using an infrared gas analyzer (LI-6400; LI-COR Inc., Lincoln, NE, USA) under photosynthetically active photon flux density of 1.800 μmol m^−2^ s^−1^ and a CO_2_ concentration in the air of 380 μmol^−1^, at a temperature of 21 to 25°C (Reis et al., 2018). The parameters measured were CO_2_ assimilation rate (A), stomatal conductance (gs), transpiration rate (E), and internal CO_2_ concentration (Ci). From these measurements, the leaf instantaneous water use efficiency (IWUE) was calculated by dividing A by E. Meanwhile, the instantaneous carboxylation use efficiency (CUE) was calculated by dividing A by Ci (Martineau et al., 2017).

For the analysis of photosynthetic pigments, chlorophyll a (Cla), b (Clb), total chlorophyll (Clt), carotenoids (Carot) and pheophytin (Pheoph) were quantified using dimethyl sulfoxide (DMSO) as the extracting agent (Hiscox and Israelstam, 1979). To characterize the stress tolerance mechanism and nitrogen metabolism, the method described by Bieleski and Turner (1966) was used. This extraction provided results for amino acids, ureides, proteins, and lipid peroxidation. The quantification of hydrogen peroxide was carried out using the methodology described by Alexieva et al. (2001).

#### 2.3.2 Components of soybean production and yield

At soybean maturity (R_8_), the following parameters were evaluated: plant population (Pop) by counting the plants in 4 meters of the four central rows of each plot, extrapolated to the number of plants per hectare; plant height (H) by measuring ten consecutive plants along the row, from the lower end in the soil to the uppermost part, with values expressed in meters (m). For evaluating the insertion height of the first pod (HFI), the same plants used to measure height were considered, from the soil to the first pod. The number of pods per plant (NPP) was measured by counting the number of pods from 10 plants per plot and calculating the average. The number of grains per plant (NGP) was determined by counting the number of grains from the same 10 plants per plot and subsequently calculating the average. Finally, the 100-grain weight (M100) was determined by separating four grain samples and taking them to the laboratory for counting with an electronic counter. The weight of the 100 grains was measured on an analytical scale (0.01 g) and the grain moisture was corrected to 13% (wet basis). Grain yield (Prod) was determined by manually harvesting the central plants of the plots (4 m from 4 rows), which were threshed and weighed for the yield per plot, with the moisture corrected to 13%, and values extrapolated to kg ha^-1^.

### 2.3 Statistical analysis

For the analyses, ten composite samples were collected from each of the 24 plots, and all 240 samples were evaluated. After analysis, the results were tabulated and the mean of the samples corresponding to each plot was calculated. Subsequently, the data from the 24 plots were analyzed (total degrees of freedom = 23; error degrees of freedom = 15). The data obtained from the analyses were tabulated and analyzed using R software, version 4.3.2 (R Core Team, 2024). Data normality was assessed using the Shapiro-Wilk test. The results were subjected to analysis of variance (ANOVA) using the F-test (*p ≤* 0.05). Regarding the presence of the commercial inoculant and the application of Mg, the data were evaluated using Tukey’s test (*p ≤* 0.05). Additionally, to improve data interpretation and correlation, a principal component analysis (PCA) was performed. For the analysis of variance, PCA construction, and development of interaction graphs in R software, the following packages were used: “ExpeDes.pt,” “FactoMineR,” “Shiny,” “FactoInvestigate,” and “ggplot2”.

## 3 Results

### 3.1 Plant physiology

#### 3.1.1 Photosynthetic pigments

The mean values for foliar spraying with Mg showed significant effects on the contents of chlorophyll a, chlorophyll b, total chlorophylls, carotenoids, and pheophytin (**Supplementary Table 1**), indicating that Mg contributed to the increase in these compounds. In the 2023/24 growing season, an increase of approximately 20% in total chlorophyll content and 16% in carotenoids was observed in the treatments that received foliar Mg application. Regarding inoculation, significant mean values were observed for pheophytin levels in the treatments with co-inoculation applied both in the seed furrow and directly on the seeds, which presented the highest values according to the statistical test (*p ≤* 0.05). Elevated pheophytin levels indicate increased chlorophyll degradation, which may negatively affect photosynthesis. It is possible that the higher mean chlorophyll content combined with high temperatures contributed to the increase in pheophytin during this season; however, this result did not negatively impact in A.

In the 2024/25 season, carotenoid content showed significant differences in response to foliar spraying, with the highest values observed in plots that received Mg application—25% higher than the control treatment without the nutrient. Meanwhile, pheophytin levels were significantly affected by both foliar spraying and inoculation. The results showed lower pheophytin contents in plots that did not receive Mg foliar application and in those inoculated via seeds. This may indicate that these plants exhibited improved metabolic performance, with reduced degradation of photosynthetic pigments under the climatic conditions of this season. Regarding the contents of photosynthetic pigments in the 2024/25 season, chlorophyll a, chlorophyll b, and total chlorophyll levels exhibited significant means for the interaction between treatments (*p ≤* 0.05).

**Figure 4:**
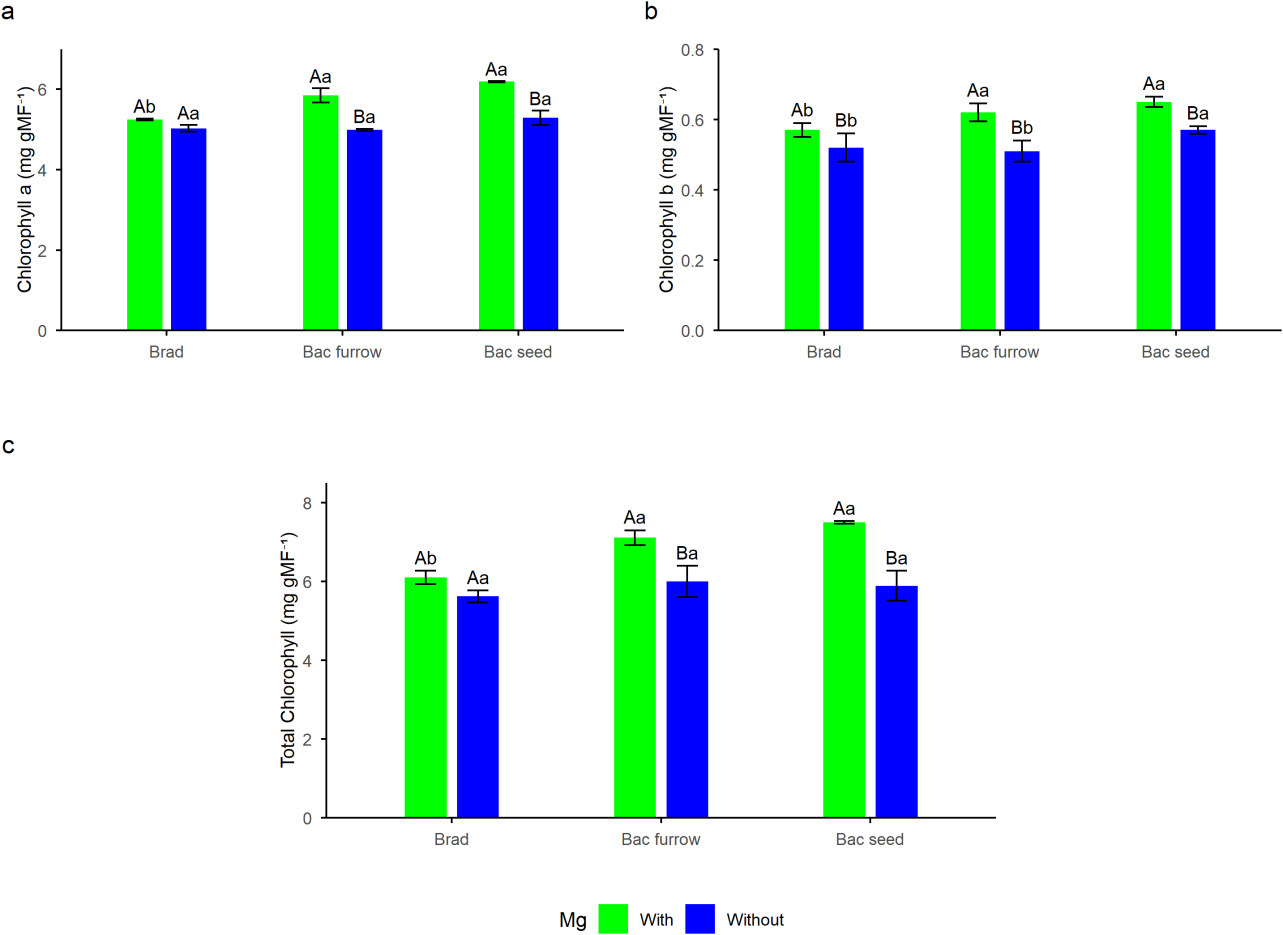
Breakdown of chlorophyll a **(a)**, chlorophyll b **(b)**, and total chlorophyll **(c)** contents in soybean under the effect of foliar Mg application and different co-inoculation arrangements with PGPR in Selvíria, state of Mato Grosso do Sul, Brazil, during the 2024/25 growing season. Means followed by the same uppercase letters within inoculation/co-inoculation and lowercase letters within Mg application do not differ from each other by the Tukey test (*p ≤* 0.05). Brad, control- application of *B. japonicum* to the seeds; Bac furrow, *B. japonicum* inoculated on the seeds and application of *B. subtilis* + *P. megaterium* in the planting furrow; Bac seed, *B. japonicum* co-inoculated with *B. subtilis* + *P. megaterium*, both applied to the seed; with, with Mg application; without, without Mg application.

The interaction effects for chlorophyll a, chlorophyll b, and total chlorophyll contents (**Figure 4**) in the 2024/25 season revealed that the combination of Mg foliar spraying with co-inoculation via seed or furrow resulted in the highest pigment contents. For chlorophyll a, the treatment with Mg application combined with furrow co-inoculation was approximately 11% higher than the control treatment (**Figure 4a**), and similar results were observed for the seed-based co-inoculation treatment, which showed chlorophyll content about 15% greater than the treatment without foliar Mg application. For chlorophyll b, the same trend was observed, with superior results in foliar Mg application for the treatments with co-inoculation via furrow and seed (**Figure 4b**), with increases of 7% and 10%, respectively.

The total chlorophyll content results showed that seed furrow and seed inoculation were superior to the control when combined with foliar Mg spraying (**Figure 4c**). Seed furrow and seed inoculation increased total chlorophyll content by approximately 14% and 18%, respectively, compared with the control. This result demonstrates that foliar Mg supply and co-inoculation were beneficial strategies for soybean, as they promoted higher levels of photosynthetic pigments even under field conditions.

#### 3.1.2 Nitrogen metabolism and stress indicators

Ureides content showed significant results (*p ≤* 0.05) for inoculation in both growing seasons (**Supplementary Table 2**). The control and seed co-inoculation treatments presented higher means compared with seed furrow co-inoculation for ureide content. The highest mean was observed in the control treatment (without co-inoculation), most likely due to potential competition among microorganisms. For Perox content in the 2024/25 season, significance was observed only for foliar spraying, with the Mg-treated plants showing lower mean values. This indicates that foliar Mg application at a “critical” stage of soybean demand may be important for reducing the levels of stress indicators even under field conditions, thereby positively influencing plant metabolism.

**Figure 5:**
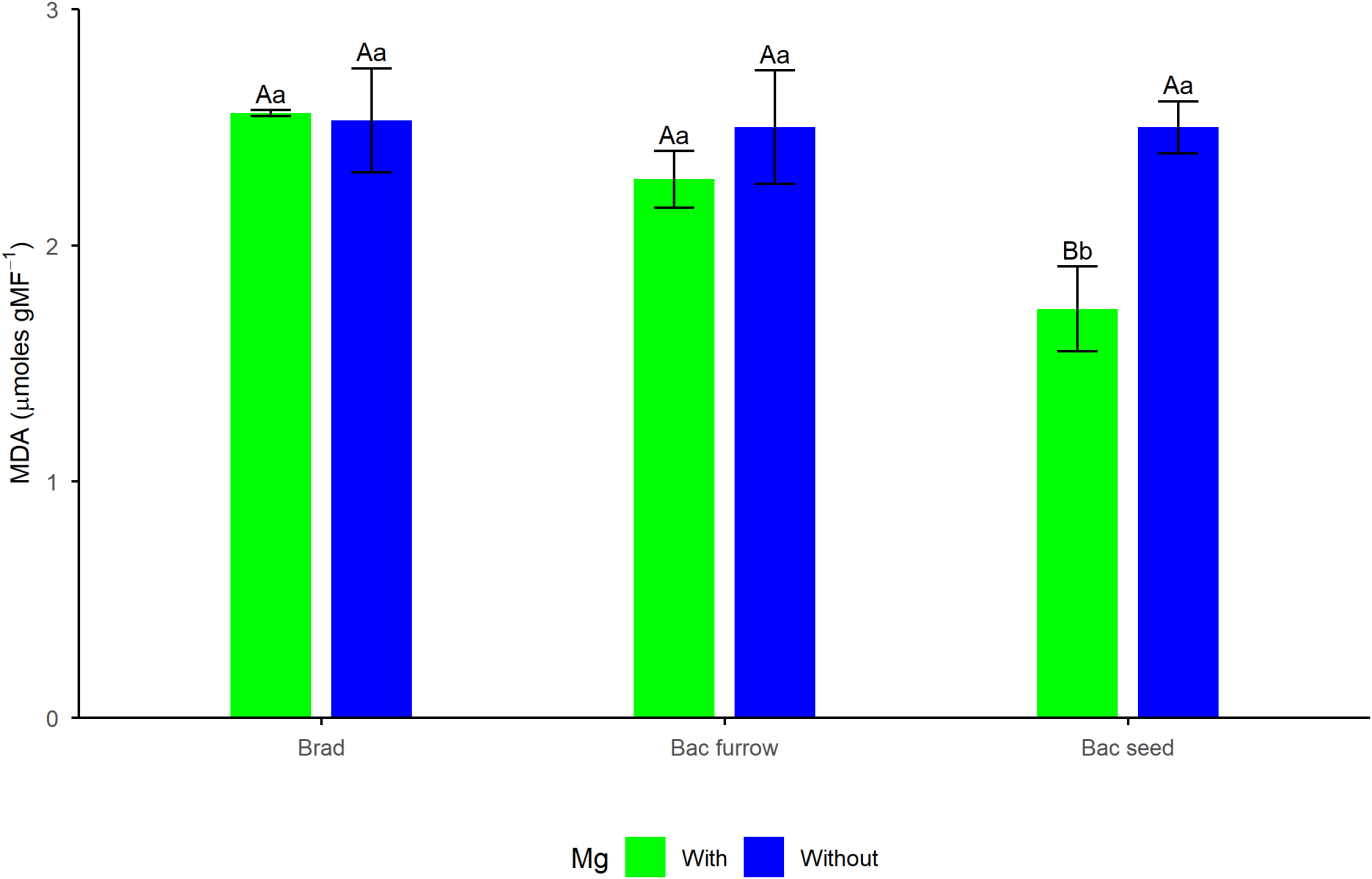
Breakdown of malondialdehyde (MDA) content in soybean under the effect of foliar Mg application and different co-inoculation arrangements with PGPR in Selvíria, state of Mato Grosso do Sul, Brazil, during the 2024/25 growing season. Means followed by the same uppercase letters within inoculation/co-inoculation and lowercase letters within Mg application do not differ from each other by the Tukey test (*p ≤* 0.05). Brad, control-application of *B. japonicum* to the seeds; Bac furrow, *B. japonicum* inoculated on the seeds and application of *B. subtilis* + *P. megaterium* in the planting furrow; Bac seed, *B. japonicum* co-inoculated with *B. subtilis* + *P. megaterium*, both applied to the seed; with, with Mg application; without, without Mg application.

Regarding AA content, descriptive statistics revealed significant differences for the isolated effects of spraying and inoculation. Mg application resulted in approximately 10% higher AA content compared with the treatment without this nutrient, whereas for inoculation, seed co-inoculation promoted a significant mean increase of 12% in AA compared with seed furrow co-inoculation, which yielded lower AA content than the control. For MDA content, the significant interaction (**Figure 5**) showed that the combination of foliar Mg spraying and seed co-inoculation resulted in the lowest MDA levels among treatments. This value was 32% lower than that of the control, indicating that foliar Mg application and the use of PGPR positively influenced the antioxidant system, reducing the levels of this oxidative stress biomarker.

#### 3.1.3 Photosynthetic metabolism

The mean values for A, Ci and IWUE were significant (*p ≤* 0.05) for the P × I interaction (spraying x inoculation) in both growing seasons (**Supplementary Table 3**). The isolated results for PGPR inoculation showed that treatments with co-inoculation via furrow and seed were able to increase E and gs indices in 2023/24. For E, the treatments showed an approximate gain of 9% compared with the control treatment. The seed co-inoculation treatment had the highest mean for gs. representing an approximate increase of 28% compared with the control.

**Figure 6:**
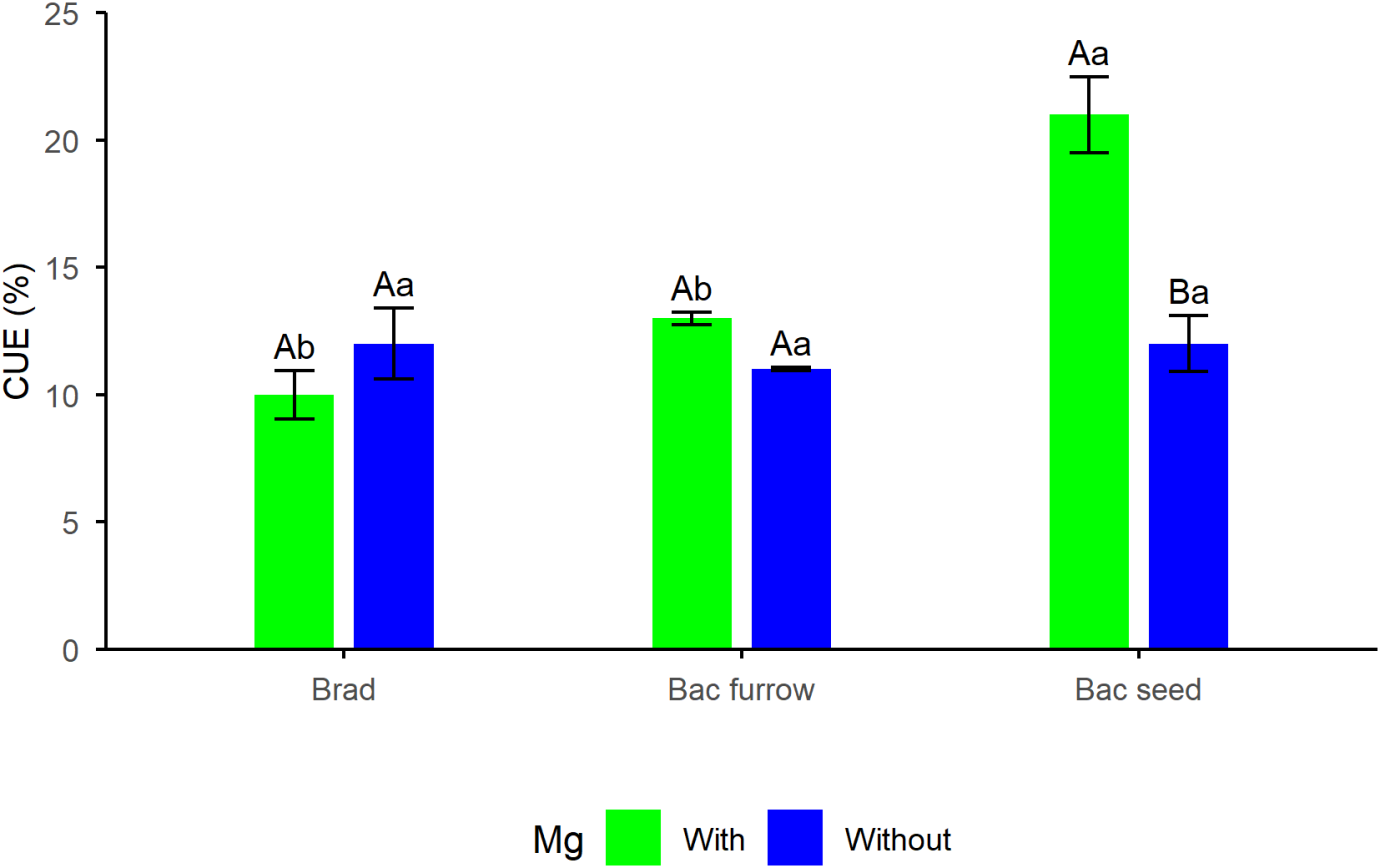
Breakdown of carboxylation efficiency (CUE) in soybean under the effect of foliar Mg application and different co-inoculation arrangements with PGPR in Selvíria, state of Mato Grosso do Sul, Brazil, during the 2023/24 growing season. Means followed by the same uppercase letters within inoculation/co-inoculation and lowercase letters within Mg application do not differ from each other by the Tukey test (*p ≤* 0.05). Brad, control-application of *B. japonicum* to the seeds; Bac furrow, *B. japonicum* inoculated on the seeds and application of *B. subtilis* + *P. megaterium* in the planting furrow; Bac seed, *B. japonicum* co-inoculated with *B. subtilis* + *P. megaterium*, both applied to the seed; with, with Mg application; without, without Mg application.

In this season, CUE presented significant means for the interaction, whereas in the 2024/25 season, the E results showed significant means for inoculation alone, with seed co-inoculation presenting the highest E. For CUE, both spraying and inoculation showed significant means for the isolated factors, with higher values for foliar Mg spraying, and in the case of PGPR, for both furrow and seed application. For CUE in the 2023/24 season (**Figure 6**), the results show that foliar Mg spraying combined with seed co-inoculation yielded the highest CUE values. An increase of 40% (compared with the control without foliar Mg application) was observed for the interaction between factors, indicating that under the conditions imposed during this season, even with high temperatures, plants were able to fix carbon effectively, which contributed to their metabolism.

**Figure 7:**
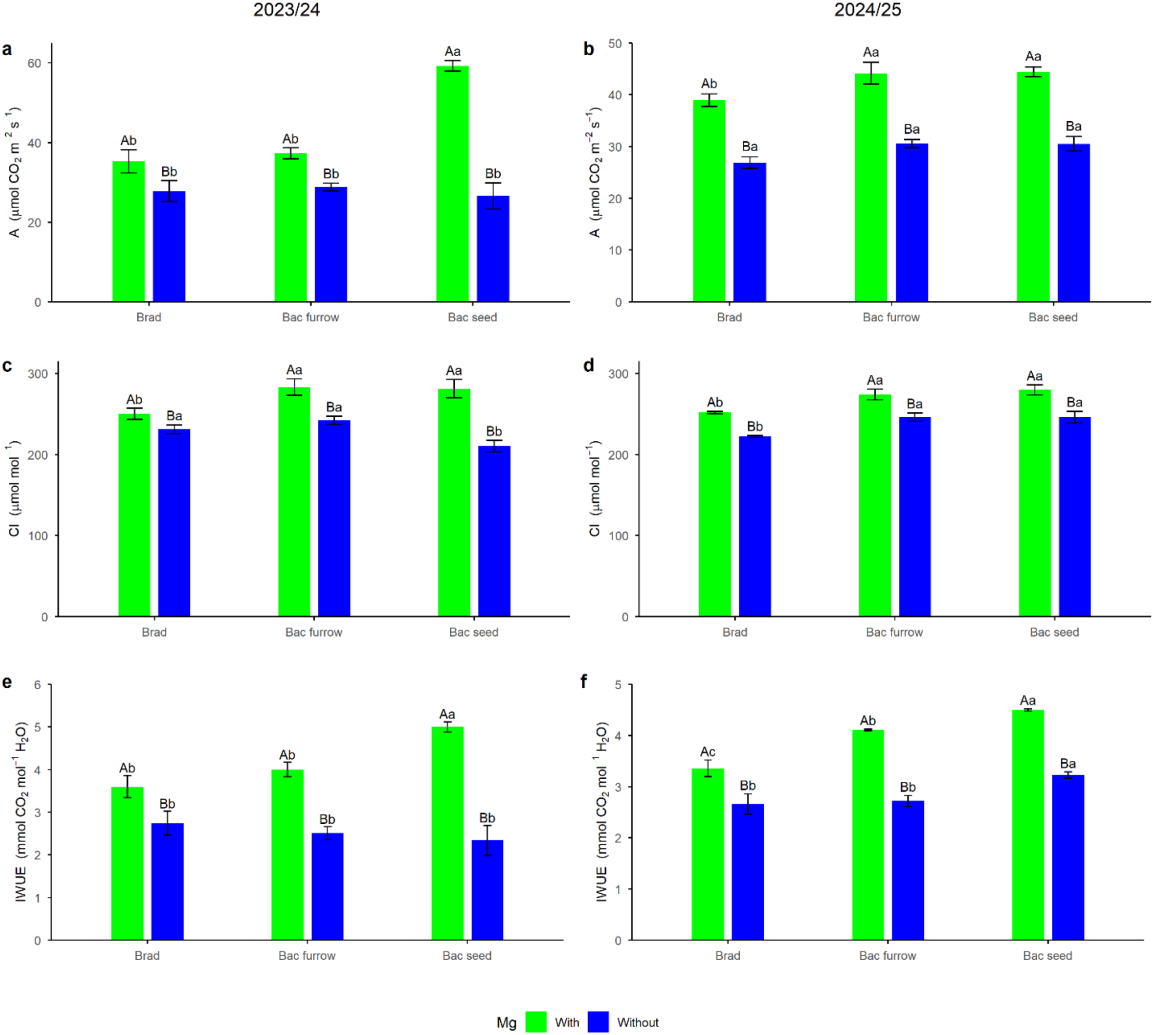
Breakdown of photosynthesis (A) **(a, b)**, internal carbon (Ci) **(c, d)**, and instantaneous water use efficiency (IWUE) **(e, f)**, in soybean under the effect of foliar Mg application and different co-inoculation arrangements with PGPR in Selvíria, state of Mato Grosso do Sul, Brazil, during the 2023/24 and 2024/25 growing seasons. Means followed by the same uppercase letters within inoculation/co-inoculation and lowercase letters within Mg application do not differ from each other by the Tukey test (*p* ≤ 0.05). Brad, control-application of *B. japonicum* to the seeds; Bac furrow, *B. japonicum* inoculated on the seeds and application of *B. subtilis* + *P. megaterium* in the planting furrow; Bac seed, *B. japonicum* co-inoculated with *B. subtilis* + *P. megaterium*, both applied to the seed; with, with Mg application; without, without Mg application.

In the 2023/24 growing season, foliar Mg spraying enhanced the effects of seed co-inoculation on A (**Figure 7a**), with a 40% increase compared with the control treatment without foliar Mg application. Similarly, to A, IWUE also benefited from foliar Mg spraying combined with seed co-inoculation, showing an increase of approximately 28% compared with the control without foliar Mg application (**Figure 7e**). This result highlights the role of internal regulation of the plant’s water content, indicating improved regulation when Mg application is combined with seed co-inoculation. The increase in this index supports the use of foliar Mg supply at critical stages of soybean development and metabolism, as well as the adoption of PGPR co-inoculation as physiological biostimulants. For Ci (**Figure 7c**), significant results were observed in the detailed analysis of co-inoculation when foliar Mg was applied, regardless of whether it was performed in the seed furrow or on the seeds. The results for A and IWUE demonstrate that, under the climatic conditions in which the experiment was conducted (**Figure 2**) during the 2023/24 season, foliar Mg application combined with seed co-inoculation was beneficial for soybean, even in soils with corrected fertility. This combination was able to considerably increase A and IWUE indices.

The breakdown results for A, Ci, and IWUE (**Figure 7**) in the 2024/25 season showed a 13% increase in A value (**Figure 7b**) with Mg application combined with either furrow or seed co-inoculation. For Ci (**Figure 7d**), the increase was approximately 10% with foliar Mg application and either furrow or seed co-inoculation, both results compared with the control without foliar Mg application. IWUE results (**Figure 7f**) indicated that only the treatment combining foliar Mg spraying with seed co-inoculation presented a significant mean among the treatments analyzed, with a 25% increase compared with the control without foliar Mg application. These results demonstrate the influence of PGPR on photosynthetic metabolism. Given the higher IWUE value and greater individual percentage increases compared with furrow application, it can be inferred that seed co-inoculation provided more promising results, with positive effects on the metabolic improvement of soybean plants in this study.

### 3.2 Soybean production and productivity

Plant height (H) and first pod insertion height (HFI) showed significant results for inoculation (*p ≤* 0.05) in the 2023/24 season (**Supplementary Table 4**). The control treatment presented the highest mean H, which can be explained by the isolated use of *Bradyrhizobium*, without competition in the rhizosphere from the PGPR provided in co-inoculation, a result that was also observed in the 2024/25 season. The lowest HFI was observed in the seed co-inoculation treatment, indicating that even with a shorter plant height, this treatment was able to promote yield gains, most likely due to enhanced root exploration and the solubilization of essential plant nutrients — both effects favored by PGPR. These nutrients, often bound in the rhizosphere, become available to plants once solubilized, thereby increasing nutrient content in plant tissues and contributing to higher photosynthetic metabolism rates, which justifies the results obtained in this study.

The Mg treatment presented the highest means for yield (Prod) (*p ≤* 0.05) in both growing seasons. On average, in the 2023/24 season there was an increase of 1092 kg ha^−1^, equivalent to approximately 18 bags, representing about a 28% yield increase solely from foliar Mg application compared with the control without Mg application. For the 2024/25 season, yield increased by 780 kg ha^-1^, or 13 bags compared to the control, in a year with high thermal fluctuations but favorable rainfall, which contributed to higher productivity. Regarding the inoculation factor, no significant means were observed in 2023/24, possibly due to the experiment being conducted in a no-till system for 13 years, suggesting that the system had reached a state of equilibrium. Nevertheless, in 2024/25, significant results were found for inoculation alone, where PGPR applied via seeds resulted in an increase of 862 kg ha^-1^, or 14 bags. These findings indicate that foliar Mg spraying in no-till areas can be a viable alternative to increase productivity. The high A values in plants receiving foliar Mg translated into significant yield gains.

**Figure 8:**
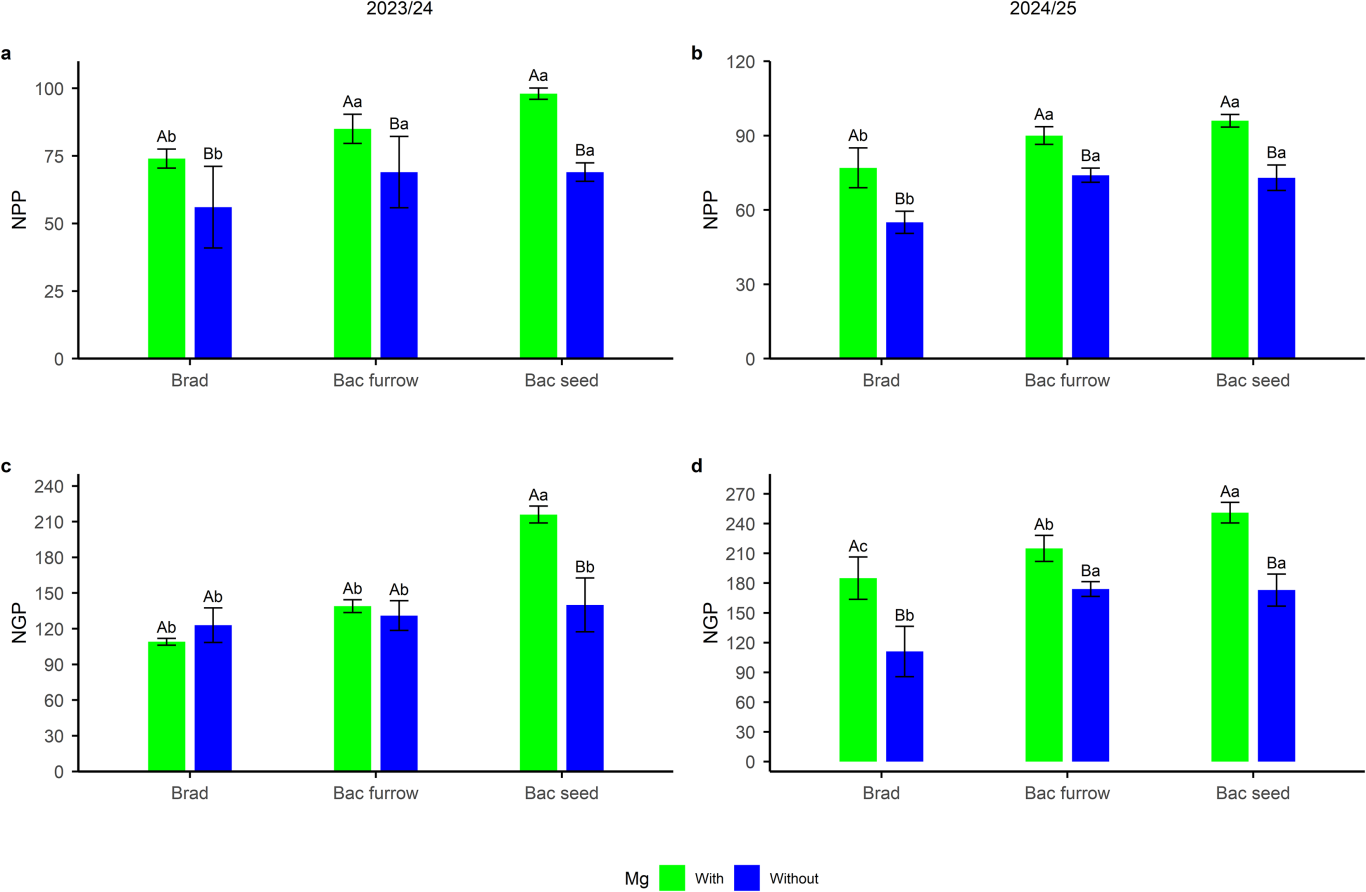
Breakdown of number of pods per plant (NPP) **(a, b)** and number of grains per plant (NGP) **(c, d)** in soybean under the effect of foliar Mg application and different co-inoculation arrangements with PGPR in Selvíria, state of Mato Grosso do Sul, Brazil, during the 2023/24 growing season. Means followed by the same uppercase letters within inoculation/co-inoculation and lowercase letters within Mg application do not differ from each other by the Tukey test (*p ≤* 0.05). Brad, control- application of *B. japonicum* to the seeds; Bac furrow, *B. japonicum* inoculated on the seeds and application of *B. subtilis* + *P. megaterium* in the planting furrow; Bac seed, *B. japonicum* co-inoculated with *B. subtilis* + *P. megaterium*, both applied to the seed; with, with Mg application; without, without Mg application.

Furthermore, the results for number of pods per plant (NPP) and number of grains per plant (NGP) showed significant means (*p *≤ 0.05) for the P × I interaction (spraying × inoculation) in both seasons. The treatment with foliar Mg application combined with seed co-inoculation resulted in higher NPP in the 2023/24 season, with an increase of 39 pods per plant — an approximate 40% increase compared with the control without foliar Mg and PGPB (**Figure 8a**). The breakdown also shows that, similar to NPP, Mg application combined with seed co-inoculation resulted in approximately 100 more grains per plant compared to the control without foliar Mg application treatment, which did not receive Mg application — an approximate increase of 45% (**Figure 8c**).

**Figure 9:**
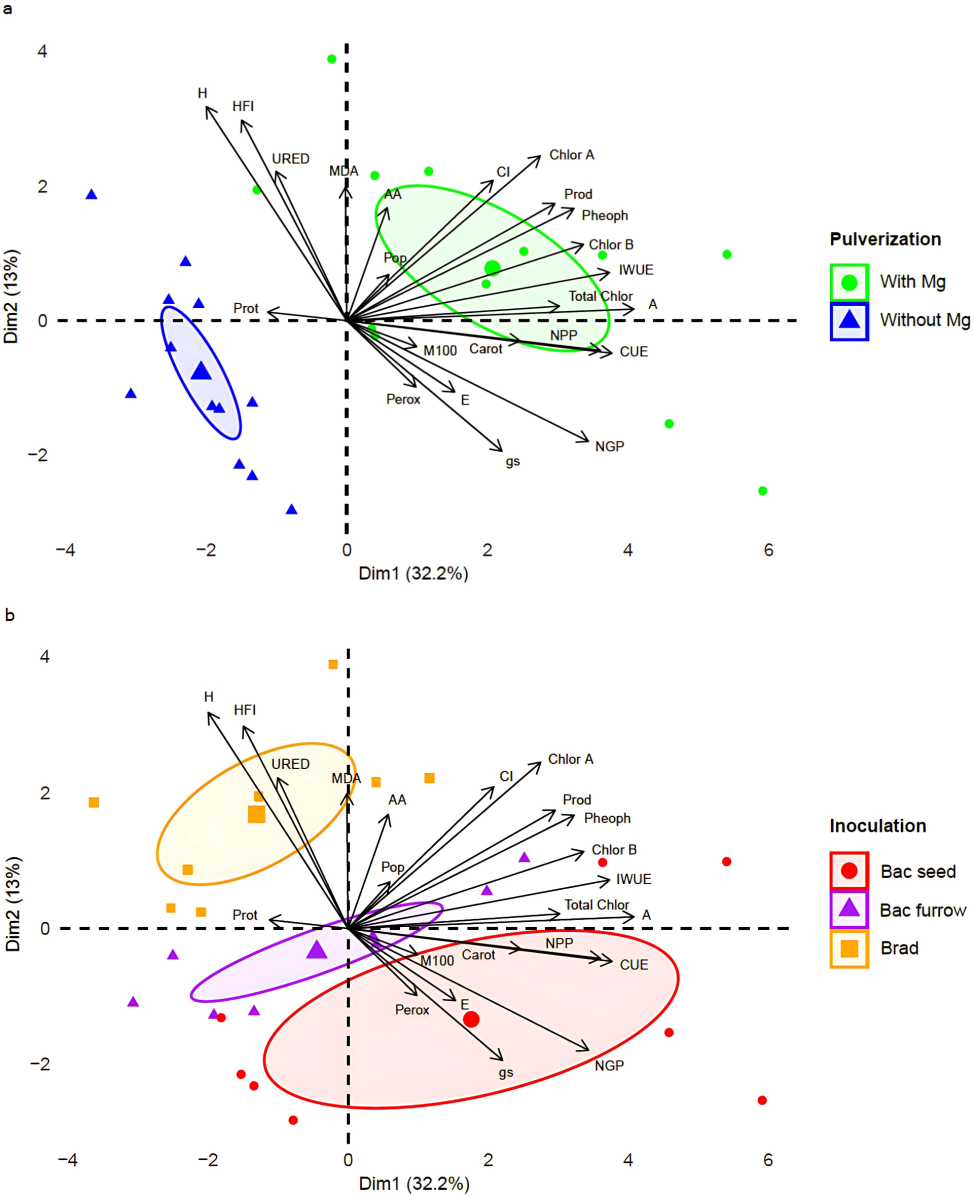
Principal component analysis (PCA) according to the presence or absence of foliar Mg application **(a)** and different co-inoculation arrangements of PGPR **(b)**, on physiological and productive parameters of soybean in Selvíria, Mato Grosso do Sul, Brazil, during the 2023/24 growing season. URED, ureides; MDA, malondialdehyde; Prot, protein; Perox, hydrogen peroxide; AA, amino acids; Chlor A, chlorophyll a; Chlor B, chlorophyll b; Chlor Total, total chlorophyll; Carot, carotenoids; Pheoph, pheophytin; A, photosynthetic rate; E, transpiration; gs, stomatal conductance; Ci, internal carbon; CUE, instantaneous carboxylation efficiency; IWUE, instantaneous water use efficiency; Pop, plant population; AltP, plant height; NGP, number of grains per plant; NVP, number of pods per plant; Prod, yield. Brad, control- application of *B. japonicum* to the seeds; Bac furrow, *B. japonicum* inoculated on the seeds and application of *B. subtilis* + *P. megaterium* in the planting furrow; Bac seed, *B. japonicum* co-inoculated with *B. subtilis* + *P. megaterium*, both applied to the seed; with, with Mg application; without, without Mg application.

The breakdown results for NPP in the 2024/25 season (**Figure 9b**) showed significant means for Mg application combined with either furrow or seed co-inoculation. A percentage increase of 16% was observed for the furrow treatment, equivalent to 13 pods, while seed co-inoculation resulted in a 20% increase, or 19 pods, compared with the control without foliar Mg and PGPB. For NGP (**Figure 9b**), the treatment with Mg application via seed inoculation presented the highest mean among the treatments. An average increase of 26%, equivalent to 66 grains, was observed compared with the control without foliar Mg and PGPB. These results demonstrate that co-inoculation with these rhizobacteria can improve plant metabolism, which may translate into gains in yield components. When combined with foliar Mg application, the results indicate an enhancement of productive gains, supporting both seed co-inoculation and foliar Mg supply as beneficial strategies.

### 3.3 Principal component analysis

#### 3.3.1 2023/24 crop season

To evaluate the relationships among the data and facilitate visualization of the contribution of the analyzed variables, Principal Component Analysis (PCA) was performed. The PCA results for the 2023/24 growing season confirmed the findings observed in the univariate descriptive statistics. Dimension 1 accounted for 32.2% of the variance, while Dimension 2 explained 13% of the variance. The PCA correlation data are presented in **Table 2**.

**Table 2:** Correlation data for principal component analysis (PCA) in soybean under presence or absence of foliar Mg spraying and PGPR inoculation, in Selvíria, state of Mato Grosso do Sul, Brazil, during the 2023/24 growing season.

PCA	PC1	PC2
Eigenvalue	7.41	3.00
Variance (%)	32.20	13.00
Cumulative variance (%)	32.20	45.20
Variables	Correlation	
URED	-0.23	0.50
MDA	0.03	0.75
Prot	-0.25	-0.04
Perox	0.23	-0.22
AA	0.13	0.38
CUE	0.85	-0.11
A	0.92	0.29
E	0.38	-0.21
gs	0.50	-0.44
Ci	0.47	0.49
IWUE	0.84	0.16
Chlor A	0.62	0.55
Chlor B	0.76	0.26
Total Chlor	0.68	0.05
Carot	0.56	-0.07
Pheoph	0.73	0.38
Pop	0.13	0.15
HFI	-0.34	0.67
H	-0.45	0.72
NGP	0.78	-0.40
NPP	0.81	0.10
M100	0.21	0.14
Prod	0.67	0.39

URED, ureides; MDA, malondialdehyde; Prot, protein; Perox, hydrogen peroxide; AA, amino acids; Chlor A, chlorophyll a; Chlor B, chlorophyll b; Chlor Total, total chlorophyll; Carot, carotenoids; Pheoph, pheophytin; A, photosynthetic rate; E, transpiration; gs, stomatal conductance; Ci, internal carbon; CUE, instantaneous carboxylation efficiency; IWUE, instantaneous water use efficiency; Pop, plant population; H, plant height; HFI, first pod insertion height; NGP, number of grains per plant; NPP, number of pods per plant; Prod, yield.

Regarding the presence or absence of foliar Mg, the PCA (**Figure 9a**) shows that the results for Mg application are located on the right side of the plot, sharing significant contributions from AA, Pop, Ci, Chlor A, Chlor B, Total Chlor, Pheoph, Prod, A, and IWUE. Notably, high correlation values were observed for A and IWUE, with respective loadings of 0.92 and 0.84 on this dimension. It is important to highlight that this dimension also exhibited a low correlation value for Perox. Additionally, low correlation values were observed for E, indicating that, due to the high IWUE values, plants well-nourished with Mg better regulate their water content. The results corresponding to the absence of foliar Mg supply are located on the left side of the plot, showing low protein (Prot) values. This outcome suggests that plants not receiving Mg were more susceptible to field stresses.

For the inoculation factor, values located on the right side of the plot represent the results of seed co-inoculation (**Figure 9b**). In this study, PGPR seed co-inoculation showed more robust results in both descriptive statistics and PCA outcomes for the 2023/24 growing season, suggesting its potential for field use. Notably, low values of E and Perox were observed, indicating that these bacteria may act on plant metabolism and contribute to the reduction of stress indicators under field conditions. The control treatment, which received only *Bradyrhizobium* inoculation, is positioned on the left side of the plot, sharing high correlations with H, HFI, URED, and MDA. These results suggest that the high MDA levels indicate that these plants were more susceptible to elevated temperatures in the field.

#### 3.3.2 2024/25 crop season

For the PCA results in the 2024/25 growing season, Dimension 1 accounted for 40.6% of the variance, while Dimension 2 explained 12% of the variance. The PCA correlation data are presented in **Table 3**.

**Table 3:** Correlation data for principal component analysis (PCA) in soybean under presence or absence of foliar Mg spraying and PGPR inoculation, in Selvíria, state of Mato Grosso do Sul, Brazil, during the 2024/25 growing season.

PCA	PC1	PC2
Eigenvalue	9.34	2.76
Variance (%)	40.60	12.00
Cumulative variance (%)	40.60	52.60
Variables	Correlation	
URED	0.07	0.59
MDA	-0.35	0.36
Prot	0.43	0.41
Perox	0.75	0.13
AA	0.52	0.21
CUE	0.89	0.20
A	0.94	-0.09
E	0.36	-0.11
gs	0.43	-0.58
Ci	0.71	-0.50
IWUE	0.86	0.04
Chlor A	0.86	0.12
Chlor B	0.84	-0.05
Total Chlor	0.87	-0.13
Carot	0.86	-0.16
Pheoph	-0.42	-0.67
Pop	0.49	0.55
HFI	-0.18	0.59
H	0.09	0.01
NGP	0.73	0.19
NPP	0.78	0.22
M100	0.17	0.09
Prod	0.64	0.39

URED, ureides; MDA, malondialdehyde; Prot, protein; Perox, hydrogen peroxide; AA, amino acids; Chlor A, chlorophyll a; Chlor B, chlorophyll b; Chlor Total, total chlorophyll; Carot, carotenoids; Pheoph, pheophytin; A, photosynthetic rate; E, transpiration; gs, stomatal conductance; Ci, internal carbon; CUE, instantaneous carboxylation efficiency; IWUE, instantaneous water use efficiency; Pop, plant population; H, plant height; HFI, first pod insertion height; NGP, number of grains per plant; NPP, number of pods per plant; Prod, yield.

**Figure 10:**
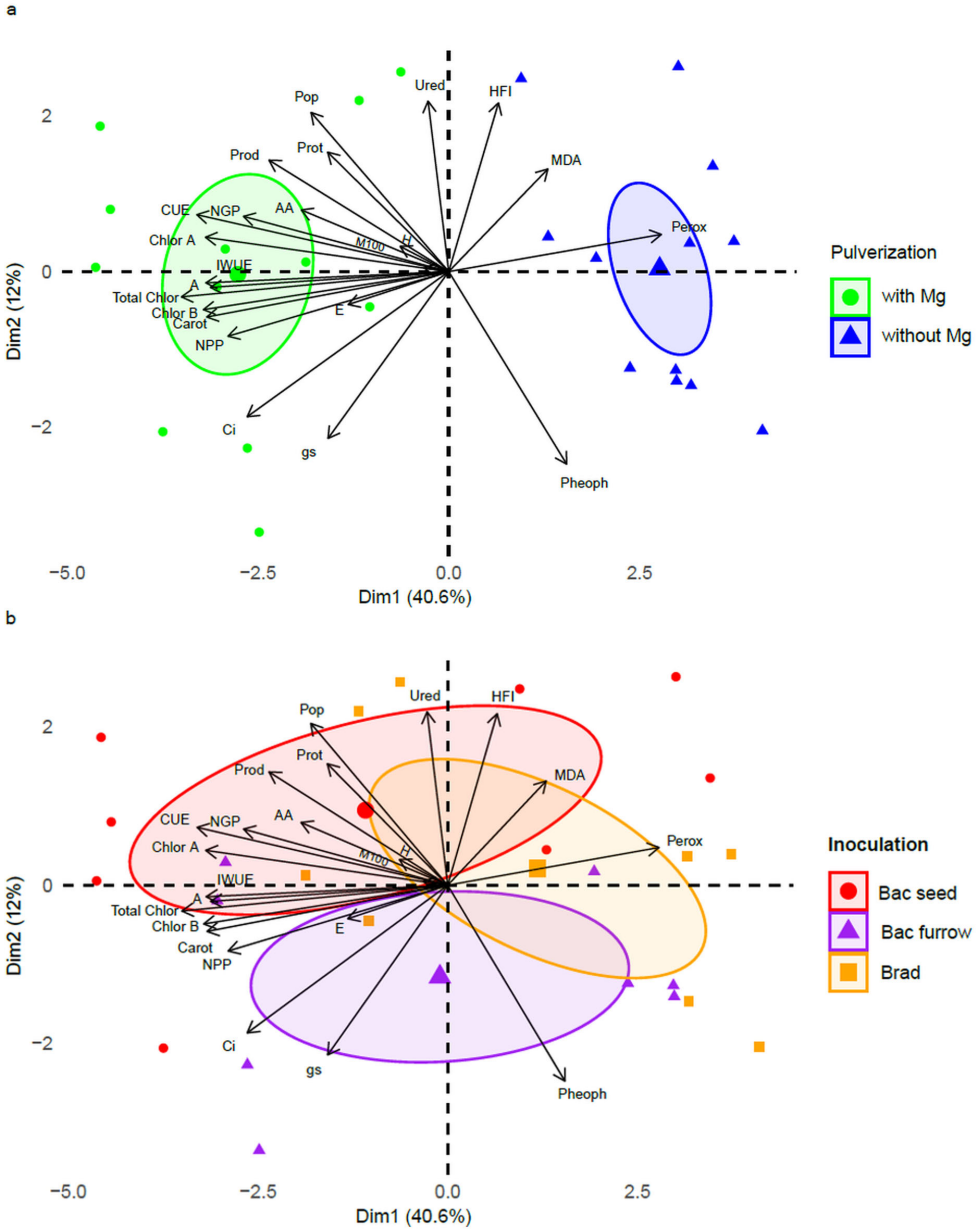
Principal component analysis (PCA) according to the presence or absence of foliar Mg application **(a)** on physiological and productive parameters of soybean, in relation to PGPR co-inoculation **(b)**, in Selvíria, state of Mato Grosso do Sul, Brazil, during the 2024/25 growing season. URED, ureides; MDA, malondialdehyde; Prot, protein; Perox, hydrogen peroxide; AA, amino acids; Chlor A, chlorophyll a; Chlor B, chlorophyll b; Chlor Total, total chlorophyll; Carot, carotenoids; Pheoph, pheophytin; A, photosynthetic rate; E, transpiration; gs, stomatal conductance; Ci, internal carbon; CUE, instantaneous carboxylation efficiency; IWUE, instantaneous water use efficiency; Pop, plant population; AltP, plant height; NGP, number of grains per plant; NVP, number of pods per plant; Prod, yield; Brad, control-application of *B. japonicum* to the seeds; Bac furrow, *B. japonicum* inoculated on the seeds and application of *B. subtilis* + *P. megaterium* in the planting furrow; Bac seed, *B. japonicum* co-inoculated with *B. subtilis* + *P. megaterium*, both applied to the seed; with, with Mg application; without, without Mg application.

The treatment without Mg application (right side of the plot) (**Figure 10a**) showed strong correlations with MDA and Perox results, indicating that plants not receiving foliar Mg were more susceptible to abiotic field stresses. Conversely, for the foliar Mg treatment (left side of the plot), variables such as Prod, AA, CUE, Chlor A, and NGP showed strong contributions to the results. Notably, the correlation values for A, CUE, Chlor A, and Total Chlor were 0.94, 0.89, 0.86, and 0.87, respectively, indicating a high contribution to the sample variance of data related to Mg application on the plant’s photosynthetic metabolism.

Regarding PGPR co-inoculation (**Figure 10b**), seed application stood out by showing higher correlations for Pop, Prot, Prod, AA, NGP, CUE, and Chlor A, indicating that this application method most positively influences physiological metabolism. Co-inoculation via furrow showed low values for Pheoph and E, which may indicate good metabolic regulation. However, low values of CI and gs suggest susceptibility to field stress. This is further corroborated by the high values of Perox and MDA in the control treatment (Brad). The high value for HFI suggests that the plant produced fewer pods, indicating that metabolism in the Brad treatment was affected by high temperatures during the experimental period, resulting in pod abortion.

According to the PCA results for both growing seasons, it is possible to infer that the data behavior changed over the two agricultural years. The 2023/24 season was affected by intense heat waves and uneven rainfall distribution, whereas in the 2024/25 season, despite high temperatures, rainfall was more evenly distributed, which contributed positively to crop development, especially during the vegetative stage. Nonetheless, variables such as Prod, Pop, AA, Chlor A, A, and IWUE, along with lower Perox levels, showed strong contributions in both years, indicating the positive effect of the treatments and demonstrating that these variables remained stable and the treatment response structure was consistent.

**Figure 11:**
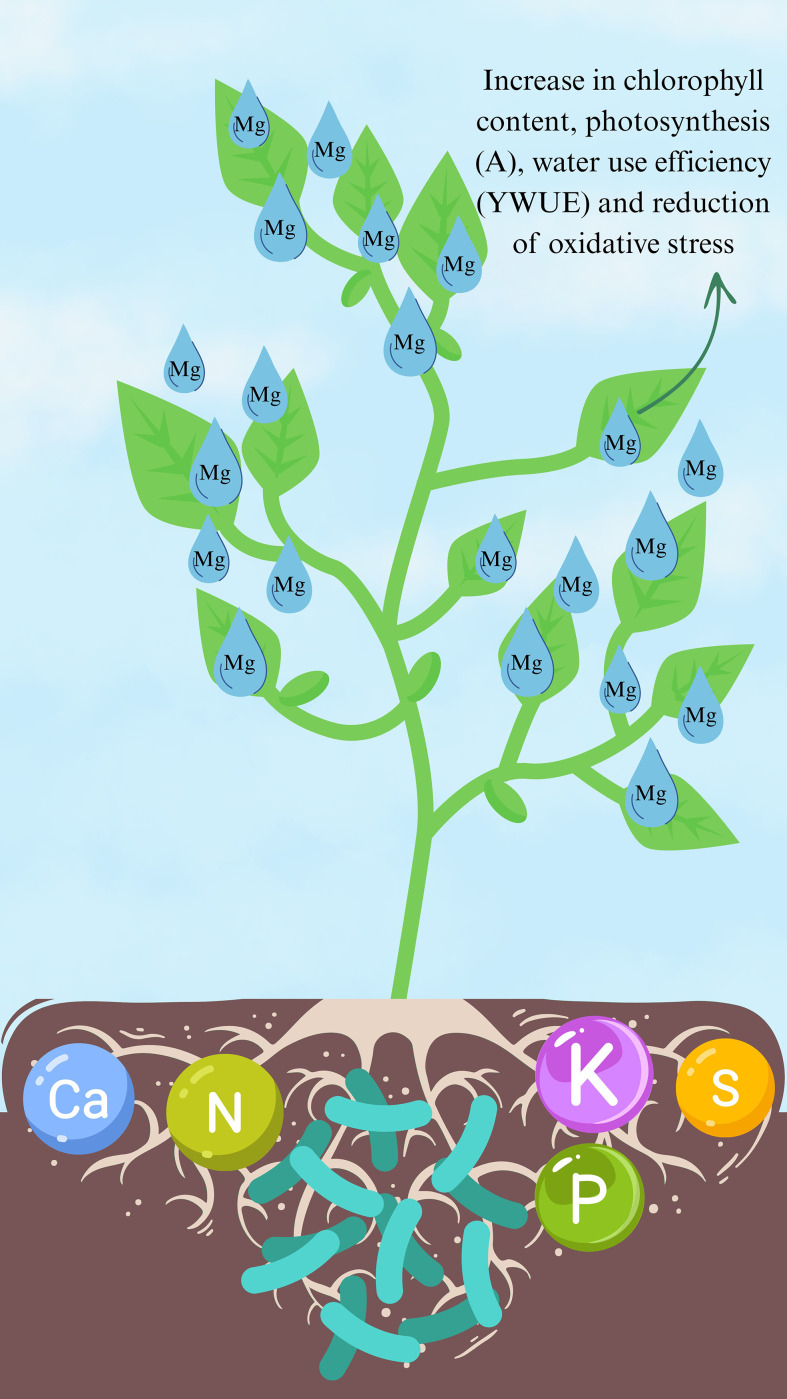
Graphical scheme of the main results observed in this study evaluating the effect of PGPR use and foliar Mg spraying as stimulators of physiological metabolism in soybean under field conditions.

What was noticeable in both years was a considerable increase in plant metabolism, particularly in foliar chlorophyll content, A, IWUE, Ci, and a decrease in oxidative compound levels. This indicates that foliar Mg spraying, even in soils under long-term no-till systems, can positively influence soybean crops, which is particularly relevant under the current climate change scenario (**Figure 11**).

## 4 Discussion

A significant improvement in plant physiology was observed in those receiving foliar Mg application, with particular emphasis on chlorophyll a and b content, total chlorophyll, carotenoids, A, Ci, and IWUE in both growing seasons (**Figures 4**, **7**). Thus, the role of this nutrient, which is the central ion of the chlorophyll molecule, became evident. Therefore, even in soils managed for years under NTS, with high base saturation and with high Mg content, foliar supply of this essential element at critical growth stages appears promising for supporting metabolism (Portis and Heldt, 1976; Cakmak, 2013), due to its stimulatory effect, considering that approximately 15 to 35% of all Mg absorbed by plants is associated with chloroplasts, mainly chlorophyll (Chen et al., 2018). Working with tomato cultivars and foliar Mg spraying, Nas and Zengin (2024) also observed an increase of about 85% in foliar chlorophyll content. For this reason, the improvement in pigment parameters observed in this study — especially chlorophyll content — is remarkable, positively reflecting on the plant’s photosynthetic physiology. An increase in pheophytin content generally signals plant stress, whereas higher chlorophyll content indicates good stability in plants with adequate Mg levels (Hamedeh et al., 2022). This highlights how foliar supplementation can be a useful strategy to support plants in the field, which are more susceptible to biotic and abiotic stresses.

Supplying Mg at key developmental stages of plants is an alternative to maintain or increase A, consequently boosting production. In this study, Mg application at the V_6_ stage of soybean resulted in a considerable increase in photosynthetic pigment content in the leaves (**Figure 4**). Wang et al. (2020) also state that Mg application can improve photosynthetic capacity, especially in photosynthetic pigment content. In their study, the authors observed that the average global gains from Mg application could lead to an average productivity increase of up to 8.5%, a figure lower than the results obtained in this work. This is due to Mg absorption via foliar application, which can be absorbed through pores in the cuticle as well as through stomata. It is noteworthy that a high foliar chlorophyll content indicates that the plant has a greater capacity for light capture, which consequently assists in water photolysis and the formation of nicotinamide adenine dinucleotide phosphate (NADP^+^). Chlorophyll is thus an essential molecule for photosynthesis, where its higher content may signify higher A, producing carbohydrates and sugars, which tend to be translated into grain yield (Chen et al., 2018; Wang et al., 2020), as observed in this study.

Supplying Mg at the V_6_ stage of soybean is important because, at this point, the plant has six nodes with fully developed trifoliate leaves, which facilitates absorption. This stage is ideal for application since, after this point, the plant may start shedding leaves, and further growth may render applications beyond this vegetative stage unfeasible. Moreover, foliar-applied Mg is metabolized more rapidly — it is estimated that within approximately 36 hours, about 60 to 80% of the applied Mg has already been absorbed and is available for plant metabolism (Dass et al., 2022). Such improvements in photosynthetic physiology justify the use of Mg in agriculture, with literature reporting yield increases of up to 25% in plants receiving foliar Mg fertilization, along with enhancements in physiological performance (Jezek et al., 2015; Salcido-Martínez et al., 2020; Raza et al., 2023; Bai et al., 2024). Thus, during critical periods of element demand, its content may be low or not readily available for specific metabolic functions. Nutrients such as calcium (Ca), Mg, and potassium (K) compete for the same absorption sites in the soil, which can affect their availability in the plant’s cellular content. In this context, foliar application emerges as an alternative to increase the concentration of this element in the plant (Briat et al., 2020). This method allows the nutrient to be more readily available and effectively support various metabolic functions, as is the case with Mg in photosynthetic parameters, as demonstrated in this study.

The plants also showed positive responses for A, Ci, and IWUE, which were enhanced by seed co-inoculation with PGPR in both growing seasons (**Figures 4**, **7**). The results for A and IWUE are particularly noteworthy and demonstrate the feasibility of foliar Mg application, even in soils with high Mg content and high base saturation —as is the case in the present study — considering that the soybean plants during the 2023/24 and 2024/25 seasons experienced critical heat wave periods with average temperatures of 37.5°C and heat indices around 47°C (**Figure 2**). Therefore, it can be affirmed that foliar Mg application and PGPR co-inoculation contributed to reducing yield losses during this period, as evidenced. In their work with wheat, Ba et al. (2020) observed that foliar application of magnesium sulfate was able to increase net photosynthesis rate, stomatal conductance, enzymatic activity, as well as nutrient accumulation and translocation to grain dry matter. These results corroborate the data obtained in this research and demonstrate the efficiency of foliar Mg use as a physiological stimulant, whose effectiveness may be further enhanced when combined with seed co-inoculation with PGPR. In our study, the plant’s ability to efficiently regulate its metabolic rates becomes evident. Even under field conditions of high temperatures during the production window (**Figure 2**), its photosynthetic apparatus ensured that the high Ci in the leaf was maintained by rubisco activity (which at elevated temperatures tends to have greater affinity for O^2^), thus preserving the plant’s energy efficiency through the use of PGPR and foliar magnesium, improving A and IWUE, and ensuring good productivity (**Figures 4**, **7**, **8**) (Chen et al., 2018; Peng et al., 2018).

Regarding IWUE, it is evident that plants receiving seed co-inoculation combined with foliar Mg application at the V_6_ stage showed superior results compared to the control (**Figure 7**). This important observation demonstrated the plant’s enhanced ability to better regulate its metabolic rates and consequently cope more effectively with stress conditions. These findings corroborate the work of Rodrigues et al. (2021), who studied soybean and maize with foliar Mg application in Mg-sufficient soils. Their study reported that foliar Mg supply resulted in increased Mg concentration in soybean (10.4%) and maize, both in the first and second seasons (13.3% and 14.4%, respectively). The authors also showed that foliar Mg application improved gas exchange performance in both crops, enhancing net photosynthesis rate and stomatal conductance by 49% and 21% in soybean, and 29% and 47% in maize, respectively. In summary, plants receiving foliar Mg were more photosynthetically efficient and better at converting carbon dioxide into carbon skeletons (sugars) — highlighted by positive CUE results in the 2023/24 season and Ci for both seasons — thereby losing less water during the process and effectively regulating gross photosynthesis rates (**Figure 6**). Studies confirm the role of Mg in regulating plant water metabolism, increasing tolerance during drought periods. These nutrient aids solute transport within plant tissues, making the proper regulation of Mg levels in tissues essential. With higher Mg content, water uptake can be regulated more efficiently (Chen et al., 2018; Peng et al., 2018; Mor et al., 2023). It is also noteworthy that PGPR from the genera *Bacillus* spp. and *Priestia* are highly effective in enhancing soil water uptake due to their ability to stimulate root growth and produce biofilms that facilitate water and nutrient absorption. Additionally, they can produce efficient phytohormones that assist in water regulation through stomatal opening and closing, supporting drought resistance (Radhakrishnan et al., 2017; Santander et al., 2024), which explains the seed co-inoculation results observed in this study.

This study found a reduction in leaf ureide content for co-inoculation treatments, both via furrow and seed. It is noteworthy that this may have occurred due to competition between the bacteria used, yet without affecting A. Studies on co-inoculation indicate that both positive and negative results may arise, depending on the bacterial strain involved, as well as on metabolism, biochemistry, type, and inoculation method (via furrow or seed), which can affect final physiological parameters and productivity (Nogueira et al., 2018; Prando et al., 2020). Nevertheless, co-inoculation showed considerable gains in overall metabolic rates, which supports the use of these bacteria.

Noteworthy are the positive results observed with co-inoculation of *Bacillus* and *Priestia* in both growing seasons (**Figure 8**). These bacteria are capable of assisting in phosphorus (P) solubilization and promoting plant growth. Studies show that both genera can form endospores, which enable them to withstand extreme abiotic conditions such as high temperatures, acidic pH, and radiation (Bahadir et al., 2018). This makes the use of these bacteria viable, especially considering the current climate change scenario that may favor their application to mitigate abiotic stresses. The study by Moretti et al. (2024), which worked with various bacterial metabolites and PGPR co-inoculation, found that it is necessary to tailor bacterial consortia according to their functionality for the specific crop. The authors also emphasize the need for further research on these bacterial consortia and the positive effects of certain bacterial groups on crop physiology and productivity. For *B. subtilis* and *P. megaterium*, Oliveira-Paiva et al. (2024), working with maize, observed an 8.9% yield increase in areas treated with *Bacillus* and *Priestia*. Regarding soybean, the authors reported an increase of about 12 to 16 bags per hectare when co-inoculated with *Bradyrhizobium* + *Bacillus* and *Priestia*. Concerning productive parameters, recent studies highlight the efficiency of *Bacillus* and *Priestia* as regulators of nutritional balance (Velloso et al., 2020; Sousa et al., 2021; Oliveira-Paiva et al., 2024), including yield increases due to their growth-promoting effects (Ribeiro et al., 2018; Guimarães et al., 2021; Leite et al., 2022; Guimarães and Klein, 2023; Oliveira-Paiva et al., 2024).

It is worth highlighting that bacteria of the genus *Bradyrhizobium* (control treatment in the present study) are Gram-negative and can be highly affected by temperature fluctuations during the production period. This finding is consistent with the results of this study, which showed lower performance for inoculation with *Bradyrhizobium* alone, especially considering the high contribution to elevated MDA and Perox levels observed in the PCA across both growing seasons (**Figures 5**, **9**, **10**). In contrast, bacteria of the genus *Bacillus* spp. are known to be Gram-positive and capable of forming endospores in the soil. These resistant structures ensure bacterial survival under adverse conditions, such as the high temperatures recorded during the experimental period (**Figure 11**) (Nogueira et al., 2018; Hungria and Nogueira, 2020; Oliveira-Paiva et al., 2024). Given that furrow inoculation faces the barrier of well-established crop residues resulting from years under NTS, its effectiveness may have been reduced. This study demonstrates that seed co-inoculation broadens the efficiency spectrum of these microorganisms, and that their combined application does not produce deleterious effects. This result is consistent with the findings of Fiuza et al. (2022), who, working with soybean, also observed greater efficiency of co-inoculation with this group of bacteria when applied via seed. The authors confirmed that nodulation efficiency, nodule dry matter, and grain yield were superior when these PGPB were applied to the seed. Several studies evaluating PGPB and application methods have shown that seed application produces better results than furrow application (Denton et al., 2017). It can be stated that such variation in outcomes depends on a series of factors intrinsic to the microorganism itself, the type of inoculant, physical barriers encountered, and soil characteristics (Lyu et al., 2021; Rigobelo et al., 2024).

Regarding grain yield, unidirectional statistical data demonstrate that the stimulatory effect induced by foliar Mg application results in positive gains in total soybean grain productivity (**Figure 8**), corroborating findings from various studies (Deliboran et al., 2011; Mor et al., 2023; Altarugio et al., 2017; Branquinho, 2020; Ba et al., 2020; Cecatto Júnior et al., 2021). In studies Peng et al. (2018) showed that adequate Mg supply to soybean can aid nodulation and consequently enhance ureide metabolism, influencing nodule formation and thus biological nitrogen fixation (BNF). Furthermore, Branquinho (2020) and Bhat et al. (2017) confirm that maintaining Mg nutritional balance is essential, as this element is indispensable for the stacking of thylakoids and the synthesis of several Calvin Cycle enzymes. A proper Mg balance ensures that the Rubisco enzyme operates more efficiently and rapidly, with a higher affinity for CO_2_. Therefore, supplying Mg as a physiological stimulant, even in soils without nutrient limitations, can modulate multiple reactions, increasing the activity of various enzymes, including Rubisco, which is fundamental for C_3_ plants. The results presented in this study demonstrate that this application broadly impacts metabolism and improves soybean grain productivity. In terms of productivity data, a significant role was observed in increasing the number of productive pods per plant (NPP) and the number of grains per plant (NGP), which were higher when seed co-inoculation was combined with foliar Mg application (**Figure 8**). Overall, field observations revealed that plants not receiving Mg foliar spraying nor co-inoculation exhibited a higher number of non-viable grains (empty pods), which was attributed to temperature fluctuations during the experimental period.

Regarding the PCA results and the lower concentration of Perox observed in the 2023/24 crop season (**Figures 9**, **10**), this outcome can be explained by improvements in photosynthetic parameters (Rodrigues et al., 2021). Concerning Mg application, literature reports benefits associated with foliar supply of this element, including the enhancement of antioxidant enzymes capable of reducing reactive oxygen species concentrations, thereby protecting cells against oxidative damage. This mechanism may have occurred in both crop seasons (Perox levels in the 2023/24 PCA and MDA levels in the descriptive statistics for 2024/25) (**Figures 5**, **9**, **10**). Additionally, there is an increase in the activity of metabolic enzymes such as catalase (CAT) and superoxide dismutase (SOD), as well as an improvement in the transport of photoassimilates to plant structures. The availability of Mg in tissues can also regulate gene expression involved in stress response (Hashem et al., 2019; Kong et al., 2020; Faizan et al., 2022; Lyu et al., 2023). These findings strongly justify the use of Mg under field conditions, especially in tropical countries, given the considerable increase in average temperatures and the rising frequency of heat waves during the soybean growing season (Chen et al., 2018; Rodrigues et al., 2021; Wang et al., 2020).

It is worth highlighting that the use of *B. subtilis* and *P. megaterium*, according to literature data, shows positive effects in improving stress conditions. This is evident from enhancements in photosystem II reactions, total photosynthesis, and stomatal conductance (Lyu et al., 2023; Romero-Munar et al., 2023). The research thus demonstrated that the combination of Mg supply with co-inoculation of these bacteria can be an important ally for effectively and sustainably increasing production.

Based on the field results, it is evident that the combination of foliar Mg application with PGPB of the genera *B. subtilis* and *P. megaterium* acts synergistically to improve soybean physiology and increase its resilience under real cultivation conditions. PGPB intensify plant metabolism by promoting greater leaf area, larger root volume, and improved nutrient uptake, while foliar Mg supply meets the resulting metabolic demand, directly contributing to physiological regulation and yield improvement. Overall, the metabolism stimulated by PGPB requires Mg for chlorophyll synthesis and stability, Rubisco activation, ATP production, and other essential functions. In addition, PGPB can reduce stress indicators by enhancing antioxidant enzyme activity, even under field conditions. Thus, the combination of PGPB and foliar Mg has a synergistic effect, protecting physiologically vulnerable sites—such as chloroplasts, stomata, membrane integrity, photosynthesis, and the antioxidant system—and ensuring higher photosynthetic efficiency and improved plant development, even under adverse conditions.

It should be noted that although environmental factors such as water deficit and extreme temperatures are uncontrollable in the field, as well as their effects on plants, the focus of this study is to improve the physiological aspects of plants through foliar Mg supply and the use of PGPR. The main findings are promising, given the reduction in Perox and MDA levels (**Figures 9**, **10**). Overall, for the co-inoculation of *B. subtilis* and *P. megaterium*, the results across the two study years are encouraging, as shown by the PCA (**Figures 9**, **10**), indicating consistent effects across different years despite varying climatic conditions. This study thus confirms the broad spectrum of actions that *B. subtilis* and *P. megaterium* genera can provide in soybean cultivation, making their use viable. Barea (2015) and Chatterjee et al. (2020) suggest that various factors mediate bacteria-plant interactions; however, understanding the mechanisms of action and signaling in the bacteria/plant association is essential to better comprehend the resulting responses. These responses are extremely important for the development of effective and, above all, sustainable agriculture with low cost.

## 5 Conclusion

Foliar application of Mg at the V_6_ stage, even in soil with adequate Mg content and managed under NTS for years, was able to improve the photosynthetic metabolism of soybean plants, resulting in gains in grain yield. The interaction between seed co-inoculation and foliar Mg application showed the best results in this study, indicating that this is the most effective method for using *B. subtilis* and *P. megaterium* in soybean cultivation. The furrow application showed intermediate results, with lower efficiency observed in soils under NTS with a high amount of surface crop residue. Foliar Mg supply and the use of PGPR promoted a considerable increase in A, chlorophyll content, and IWUE, as well as an increase in the number of grains and pods per soybean plant. This effect is particularly relevant in the current context, making further studies essential to understand these responses under different edaphoclimatic conditions and various limiting factors. Regarding the decrease in Perox and MDA levels observed in the PCA and descriptive statistics in this study, it is important to highlight that these results are promising and deserve focus across different crops and production systems, given the current scenario of global climate change, which places food security at risk. Such findings are encouraging, since controlling stress under field conditions is challenging. Understanding the dynamics and effects of foliar Mg supplementation and PGPR becomes essential and may ensure the continuity of agricultural production in the coming years.

## Data Availability

The datasets presented in this study can be found in online repositories. The names of the repository/repositories and accession number(s) can be found in the article/**Supplementary Material**.
