# Supplementary material for: Foliar application of magnesium and the use of plant growth-promoting rhizobacteria improve photosynthetic physiology and the yield components of soybean
Source: Front Plant Sci. 2026 Jan 20;16:1694929. doi: 10.3389/fpls.2025.1694929 (PMC12866984; doi:10.3389/fpls.2025.1694929)
Supplement: Supplementary file 1 [file Table1.docx]

**SM Table 1:** Analysis of variance for the variables chlorophyll a, chlorophyll b, total chlorophylls, carotenoids, and pheophytin in soybean crops as a function of PGPR and foliar Mg application, in Selvíria, MS, Brazil, 2023/24 and 2024/25 growing seasons.

|  | **Chlorophyll a** | | **Chlorophyll b** | | **Total Chlorophyll** | | **Carotenoids** | | **Pheophytin** | |
| --- | --- | --- | --- | --- | --- | --- | --- | --- | --- | --- |
|  | mg gMF^-1^ | | mg gMF^-1^ | | mg gMF^-1^ | | mg gMF^-1^ | | index | |
|  | Pulverization (P) | | | | | | | | |  |
|  | 2023/24 | 2024/25 | 2023/24 | 2024/25 | 2023/24 | 2024/25 | 2023/24 | 2024/25 | 2023/24 | 2024/25 |
| with Mg | 6.31 a | 5.11 | 0.85 a | 0.64 | 7.36 a | 6.89 | 0.74 a | 0.56 a | 1.53 a | 1.40 b |
| without Mg | 5.27 b | 5.80 | 0.61 b | 0.54 | 5.94 b | 5.84 | 0.62 b | 0.42 b | 1.44 b | 1.42 a |
|  | Inoculation (I) | | | | | | | | |  |
| control | 5.80 | 5.15 | 0.68 | 0.56 | 6.60 | 5.85 | 0.64 | 0.48 | 1.44 b | 1.41 a |
| furrow | 5.50 | 5.42 | 0.75 | 0.59 | 6.55 | 6.56 | 0.68 | 0.49 | 1.52 a | 1.42 a |
| seed | 5.88 | 5.74 | 0.75 | 0.6 | 6.79 | 6.70 | 0.71 | 0.49 | 1.50 ab | 1.39 b |
|  | Values of Pr>Fc | | | | | | | | |  |
| Pulverization | 0.001* | 0.001* | 0.001* | 0.001* | 0.001* | 0.001* | 0.02* | 0.001* | 0.001* | 0.01* |
| Inoculation | 0.06^ns^ | 0.001* | 0.22^ns^ | 0.06^ns^ | 0.62^ns^ | 0.003* | 0.42^ns^ | 0.81^ns^ | 0.03* | 0.06* |
| P x I | 0.80^ns^ | 0.004* | 0.37^ns^ | 0.02* | 0.63^ns^ | 0.05* | 0.91^ns^ | 0.16^ns^ | 0.88^ns^ | 0.06^ns^ |
| **Overall Mean** | 5.8 | 5.43 | 0.73 | 0.59 | 6.65 | 6.40 | 0.68 | 0.49 | 1.48 | 1.41 |
| **LSD** | 0.37 | 0.26 | 0.08 | 0.04 | 0.43 | 0.55 | 0.09 | 0.06 | 0.05 | 0.01 |
| **CV(%)** | 7.33 | 3.65 | 13.34 | 3.61 | 7.40 | 6.84 | 14.99 | 20.20 | 3.72 | 1.02 |

*Significant at p ≤ 0.05; ns: not significant at p ≤ 0.05; Mg: magnesium; control: *B. japonicum*; furrow: *Bradyrhizobium japonicum* + combination of *B. subtilis* and *P. megaterium* applied in the furrow; seed: *B. japonicum* + combination of *B. subtilis* and *P. megaterium* applied to the seeds; Means followed by the same letter, within spraying (P) and inoculation (I), do not differ statistically at the 5% significance level; LSD: least significant difference; CV: coefficient of variation.

**SM Table 2:** Analysis of variance for the variables ureides, malondialdehyde (MDA), protein, peroxide and amino acids (AA) in soybean crops as a function of PGPR and foliar Mg application, in Selvíria, MS, Brazil, 2023/24 and 2024/25 growing seasons.

|  | **Ureides** | | **MDA** | | **Protein** | | **Perox** | | **AA** | |
| --- | --- | --- | --- | --- | --- | --- | --- | --- | --- | --- |
|  | µmoles gMF^-1^ | | µmoles gMF^-1^ | | µmoles gMF^-1^ | | µmoles gMF^-1^ | | µmoles gMF^-1^ | |
|  | Spraying (S) | | | | | | | | | |
|  | 2023/24 | 2024/25 | 2023/24 | 2024/25 | 2023/24 | 2024/25 | 2023/24 | 2024/25 | 2023/24 | 2024/25 |
| with Mg | 2.40 | 2.40 | 3.10 | 2.26 | 3.70 | 3.40 | 1.10 | 0.78 b | 22.20 | 21.12 b |
| without Mg | 2.60 | 2.10 | 2.90 | 2.43 | 3.80 | 3.60 | 0.70 | 1.72 a | 20.50 | 23.25 a |
|  | Inoculation (I) | | | | | | | | | |
| control | 3.30 a | 2.91 a | 3.20 | 2.54 | 3.60 | 3.37 | 0.70 | 1.29 | 21.60 | 22.24 ab |
| furrow | 1.70 b | 1.60 b | 2.90 | 2.38 | 4.20 | 3.43 | 0.85 | 1.29 | 21.50 | 20.73 b |
| seed | 2.50 ab | 2.23 ab | 3.00 | 2.11 | 3.40 | 3.67 | 1.24 | 1.16 | 20.90 | 23.59 a |
|  | Values of Pr>Fc | | | | | | | | | |
| Spraying | 0.52^ns^ | 0.22^ns^ | 0.29^ns^ | 0.28^ns^ | 0.67^ns^ | 0.13^ns^ | 0.25^ns^ | 0.001* | 0.22^ns^ | 0.005* |
| Inoculation | 0.006* | 0.005* | 0.41^ns^ | 0.11^ns^ | 0.08^ns^ | 0.27^ns^ | 0.43^ns^ | 0.50^ns^ | 0.89^ns^ | 0.009* |
| S×I | 0.89^ns^ | 0.53^ns^ | 0.88^ns^ | 0.04* | 0.59^ns^ | 0.97^ns^ | 0.08^ns^ | 0.74^ns^ | 0.95^ns^ | 0.91^ns^ |
| **Overall Mean** | 2.50 | 2.24 | 3.02 | 2.34 | 3.70 | 3.49 | 0.90 | 1.25 | 21.3 | 22.18 |
| **LSD** | 0.74 | 0.69 | 0.42 | 0.49 | 0.59 | 0.33 | 0.85 | 0.20 | 2.92 | 2.02 |
| **CV(%)** | 34.40 | 23.65 | 16.30 | 16.32 | 18.5 | 10.87 | 10.00 | 18.94 | 15.70 | 7.15 |

*Significant at p ≤ 0.05; ns: not significant at p ≤ 0.05; Mg: magnesium; control: *B. japonicum*; furrow: *Bradyrhizobium japonicum* + combination of *B. subtilis* and *P. megaterium* applied in the furrow; seed: *B. japonicum* + combination of *B. subtilis* and *P. megaterium* applied to the seeds; Means followed by the same letter, within spraying (P) and inoculation (I), do not differ statistically at the 5% significance level; LSD: least significant difference; CV: coefficient of variation.

**SM Table 3:** Analysis of variance for the variables photosynthetic rate (A), transpiration (E), stomatal conductance (GS), internal carbon (Ci), instantaneous water-use efficiency (IWUE), and carboxylation-use efficiency (CUE) in soybean crops as a function of PGPR and foliar Mg application, in Selvíria, MS, Brazil, 2023/24 and 2024/25 growing seasons.

|  | **A** | | **E** | | **GS** | | **Ci** | | **EIUA** | | **CUE** | |
| --- | --- | --- | --- | --- | --- | --- | --- | --- | --- | --- | --- | --- |
|  | µmol CO_2_ m^-2^ s^-1^ | | mmol H_2_O m^-2^ s^-1^ | | mmol H_2_O m^-2^ s^-1^ | | µmol mol^-1^ | | mmol CO_2_ mol^-1^ H_2_O | | % | |
|  | Spraying (S) | | | | | | | | |  |  |  |
|  | 2023/24 | 2024/25 | 2023/24 | 2024/25 | 2023/24 | 2024/25 | 2023/24 | 2024/25 | 2023/24 | 2024/25 | 2023/24 | 2024/25 |
| with Mg | 44.00 | 42.52 | 11.10 | 9.94 | 1167.67 | 1286.33 a | 279.83 | 271.92 | 4.00 | 4.20 | 11 | 15 a |
| without Mg | 27.70 | 28.68 | 11.70 | 10.13 | 1111.08 | 1094 b | 237.58 | 234.67 | 2.40 | 2.87 | 16 | 12 b |
|  | Inoculation (I) | | | | | | | | |  |  |  |
| control | 31.50 | 32.92 | 10.40 b | 9.67 b | 949.50 b | 1113.75 | 245.5 | 236.88 | 2.90 | 3.38 | 11 | 12 b |
| furrow | 33.10 | 36.37 | 11.30 ab | 9.83 b | 1152.60 ab | 1196.25 | 262.62 | 260.00 | 3.20 | 3.80 | 12 | 14 a |
| seed | 43.00 | 37.50 | 11.70 a | 10.59 a | 1316.00 a | 1260.50 | 268.00 | 262.75 | 3.70 | 4.10 | 16 | 14 a |
|  | Values of Pr>Fc | | | | | | | | |  |  |  |
| Spraying | 0.0001* | 0.0001* | 0.85^ns^ | 0.21^ns^ | 0.51^ns^ | 0.005* | 0.0001* | 0.001* | 0.0001* | 0.0001* | 0.001* | 0.0001* |
| Inoculation | 0.0001* | 0.0029* | 0.026* | 0.0003* | 0.009* | 0.16^ns^ | 0.08^ns^ | 0.01* | 0.02* | 0.002* | 0.01* | 0.0003* |
| S×I | 0.0001* | 0.03* | 0.48^ns^ | 0.11^ns^ | 0.06^ns^ | 0.11^ns^ | 0.04* | 0.002* | 0.002* | 0.02* | 0.004* | 0.56ns |
| **Overall Mean** | 35.90 | 35.60 | 11.10 | 10.03 | 1139.4 | 1190.17 | 258.7 | 253.30 | 3.30 | 3.54 | 13 | 14 |
| **LSD** | 3.21 | 2.95 | 0.79 | 0.79 | 117.00 | 127.00 | 17.12 | 14.28 | 0.41 | 0.27 | 1.40 | 1.00 |
| **CV(%)** | 10.30 | 6.38 | 8.30 | 3.60 | 17.90 | 12.27 | 7.60 | 4.34 | 14.50 | 5.97 | 12.47 | 5.87 |

*Significant at p ≤ 0.05; ns: not significant at p ≤ 0.05; Mg: magnesium; control: *B. japonicum*; furrow: *Bradyrhizobium japonicum* + combination of *B. subtilis* and *P. megaterium* applied in the furrow; seed: *B. japonicum* + combination of *B. subtilis* and *P. megaterium* applied to the seeds; Means followed by the same letter, within spraying (P) and inoculation (I), do not differ statistically at the 5% significance level; LSD: least significant difference; CV: coefficient of variation.

**SM Table 4:** Analysis of variance for the variables plant height (H), first pod insertion height (HFI), plant population (Pop), number of pods per plant (NPP), number of grains per pod (NGP), 100-grain weight (M100), and grain yield (Prod) in soybean crops as a function of PGPR and foliar Mg application, in Selvíria, MS, Brazil, 2023/24 and 2024/25 growing seasons.

|  | **H** | | **HFI** | | **Pop** | | **NPP** | | **NGP** | | **M100** | | **Prod** | |
| --- | --- | --- | --- | --- | --- | --- | --- | --- | --- | --- | --- | --- | --- | --- |
|  | m | | m | | Plants ha^-1^ x 1000 | |  | |  | | g | | kg ha^-1^ | |
|  | Spraying (S) | | | | | | | | | | | | |  |
|  | 2023/24 | 2024/25 | 2023/24 | 2024/25 | 2023/24 | 2024/25 | 2023/24 | 2024/25 | 2023/24 | 2024/25 | 2023/24 | 2024/25 | 2023/24 | 2024/25 |
| with Mg | 1,30 | 1,30 | 0,33 | 0,24 | 169,80 | 181,00 | 83 | 90 | 155 | 215 | 17,19 | 14,25 | 3792 a | 4560 a |
| without Mg | 1,30 | 1,30 | 0,33 | 0,25 | 161,40 | 161,11 | 69 | 68 | 131 | 152 | 16,75 | 13,75 | 2700 b | 3780 b |
|  | Inoculation (I) | | | | | | | | | | | | |  |
| control | 1,36 a | 1,35 a | 0,34 a | 0,24 | 163,90 | 168,05 | 70 | 67 | 116 | 155 | 17,04 | 14,00 | 3056 | 3828 b |
| furrow | 1,30 b | 1,25 b | 0,33 a | 0,22 | 172,20 | 162,77 | 69 | 84 | 135 | 190 | 16,93 | 13,75 | 3285 | 3993 ab |
| seed | 1,24 c | 1,27 ab | 0,30 b | 0,25 | 160,80 | 181,66 | 89 | 84 | 178 | 204 | 16,94 | 14,30 | 3397 | 4690 a |
|  | Values of Pr>Fc | | | | | | | | | | | | |  |
| Pulverization | 0,54^ns^ | 0,10^ns^ | 0,58^ns^ | 0,36^ns^ | 0,53^ns^ | 0,1^ns^ | 0,0046* | 0,0001* | 0,03* | 0,005* | 0,46^ns^ | 0,15^ns^ | 0,001* | 0,004* |
| Inoculation | 0,001* | 0,01* | 0,001* | 0,22^ns^ | 0,76^ns^ | 0,5^ns^ | 0,002* | 0,007* | 0,0005* | 0,03* | 0,98^ns^ | 0,30^ns^ | 0,44^ns^ | 0,001* |
| S×I | 0,30^ns^ | 0,54^ns^ | 0,34^ns^ | 0,30^ns^ | 0,15^ns^ | 0,28^ns^ | 0,004* | 0,008* | 0,006* | 0,03* | 0,11^ns^ | 0,12^ns^ | 0,30^ns^ | 0,89^ns^ |
| **Overall Mean** | 1,30 | 1,30 | 0,33 | 0,24 | 165,60 | 170,8 | 76 | 79 | 143 | 183 | 16,97 | 14,00 | 3246 | 4170 |
| **LSD** | 0,031 | 0,05 | 0,015 | 0,026 | 27,66 | 21,39 | 9,28 | 13 | 21,74 | 45,40 | 1,25 | 1,06 | 462,18 | 730,2 |
| **CV(%)** | 2,80 | 4,53 | 5,50 | 12,39 | 19,20 | 9,64 | 14,00 | 13,06 | 17,50 | 19,03 | 8,50 | 5,88 | 16,40 | 13,47 |

*Significant at p ≤ 0.05; ns: not significant at p ≤ 0.05; Mg: magnesium; control: *B. japonicum*; furrow: *Bradyrhizobium japonicum* + combination of *B. subtilis* and *P. megaterium* applied in the furrow; seed: *B. japonicum* + combination of *B. subtilis* and *P. megaterium* applied to the seeds; Means followed by the same letter, within spraying (P) and inoculation (I), do not differ statistically at the 5% significance level; LSD: least significant difference; CV: coefficient of variation.
